# Proteomic profiling of plasma extracellular vesicles reveals a therapeutically targetable liver-heart axis in cardiac transplantation

**DOI:** 10.1172/jci.insight.200422

**Published:** 2026-07-02

**Authors:** Shiyu Dai, Wei Zhou, Fangyu Chen, Huanyu Zhang, Zhenchun Ji, Xuejing Zong, Wanruo Zhang, Jie Hu, Shumin Jiang, Fei Wang, Zhenya Shen

**Affiliations:** 1 Institute for Cardiovascular Science & Department of Cardiovascular Surgery of the First Affiliated Hospital, Soochow University, Suzhou, Jiangsu, China.

**Keywords:** Cardiology, Immunology, Cardiovascular disease, Immunotherapy, Transplantation

## Abstract

Extracellular vesicle–mediated interorgan communication represents a promising frontier in transplant immunology; however, its role in cardiac allograft rejection remains poorly characterized. We performed proteomic profiling of plasma-derived extracellular vesicles in a rat heterotopic heart transplantation model and identified a distinct liver-predominant protein signature during acute rejection, with antithrombin III (ATIII) emerging as a top candidate. Functional validation revealed that pharmacological extracellular vesicle inhibition intensified systemic and intragraft inflammation, whereas adeno-associated virus–mediated silencing of hepatic ATIII directly accelerated allograft rejection. Conversely, adeno-associated virus–mediated hepatocyte-specific ATIII overexpression attenuated rejection pathology, reduced immune cell recruitment, and markedly prolonged median graft survival. This protective effect was achieved without evidence of coagulopathic complications, indicating an immunomodulatory mechanism beyond ATIII’s canonical anticoagulant function. Mechanistically, ATIII overexpression was associated with upregulation of heme oxygenase-1 (HO-1) in the liver and suppression of proinflammatory cytokine expression in the graft. These findings highlight hepatocyte-derived extracellular vesicles as important mediators of a liver-heart signaling axis in transplant rejection and further implicate the protein ATIII as a contributor to this axis. Our study reveals a therapeutically targetable liver-heart signaling axis in transplant rejection, whereby enhancing liver-derived ATIII or its downstream pathways (such as HO-1) could attenuate acute cardiac allograft rejection.

## Abstract 2

Liver-derived extracellular vesicles carrying ATIII reduce inflammation and protect heart transplants from rejection.

## Graphical abstract

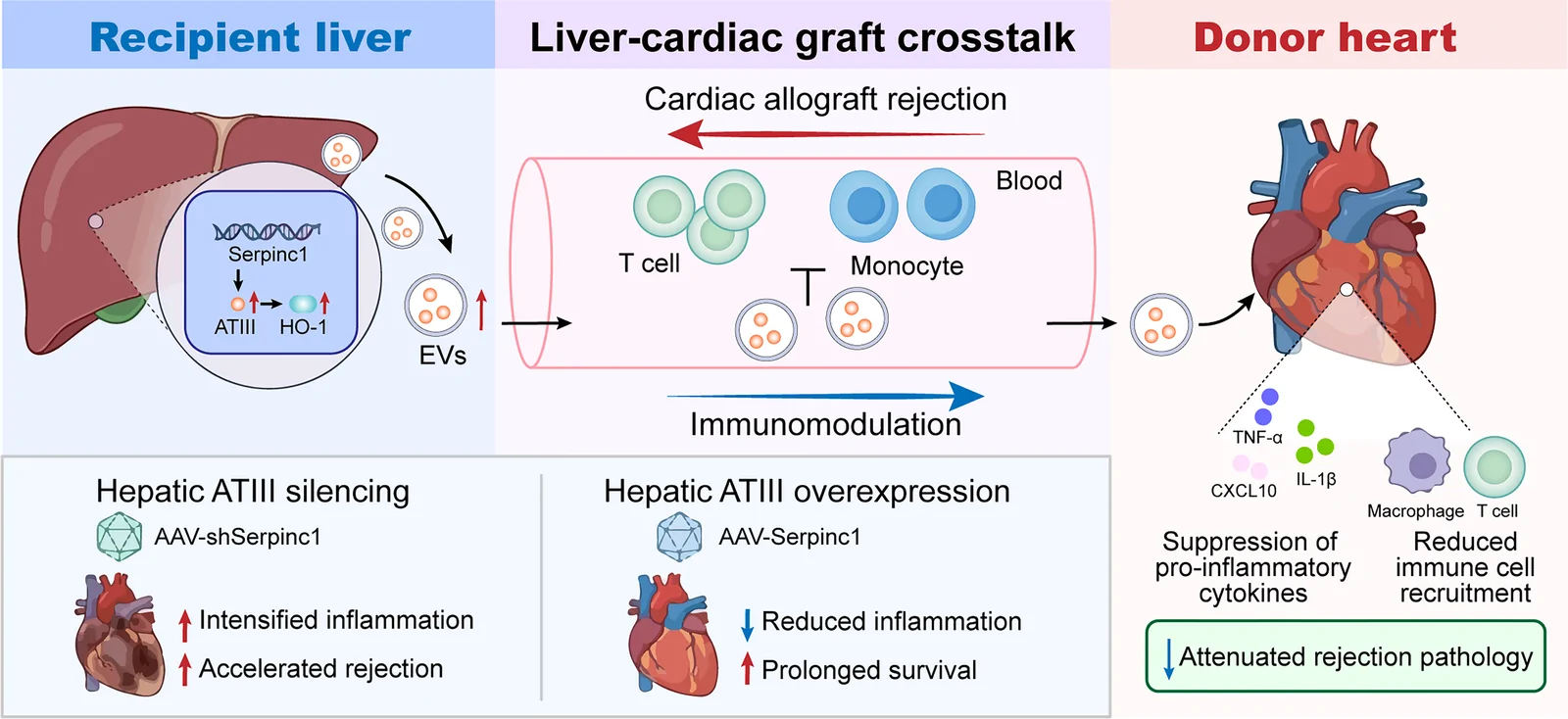

## Introduction

Heart transplantation is a life-saving intervention for end-stage heart failure, yet acute rejection remains a critical barrier to long-term graft survival (1). Current therapies rely on systemic immunosuppression, which can be effective but carries considerable side effects and variable efficacy among individuals. There is a pressing need for novel strategies that target alternative pathways involved in graft rejection while minimizing generalized immunosuppression.

Interorgan communication networks are fundamental to maintaining immune and tissue homeostasis (2, 3). Emerging evidence indicates that transplant immunobiology extends beyond the graft and lymphoid organs alone; nonlymphoid organs can profoundly influence alloimmune response through circulating factors. For instance, the liver and gut have been implicated in modulating transplant outcomes via metabolic and immunological signals. Specifically, hepatic PCSK9/CD36 signaling reprograms cardiac allograft macrophages, and PCSK9 ablation reduces rejection severity (4). On the other hand, alterations in gut microbiome composition and function may affect renal transplant rejection (5), while gut microbiome–derived metabolites promote renal allograft tolerance by inducing Tregs (6). Although these systemic interactions are increasingly recognized, the specific molecular vehicles that facilitate this long-distance organ-to-graft dialogue remain poorly defined.

Extracellular vesicles (EVs) have garnered intense interest as mediators of intercellular and interorgan communication in various pathophysiological conditions. EVs orchestrate biological processes such as immune modulation and inflammation through targeted delivery of proteins, miRNAs, and lipids (7–11). The contents of EVs reflect the characteristics and metabolic status of their cellular or tissue origins, as well as alterations in the cellular microenvironment, thereby making them valuable indicators of cellular and tissue status in various diseases (12). Notably, the circulating EV proteome dynamically reflects graft-host interactions, enabling noninvasive monitoring of acute rejection (13). However, the functional hierarchy and tissue origins of rejection-associated EV proteins in cardiac transplantation remain unexplored. Critically, the role of nonlymphoid organs (e.g., liver, brain) in coordinating alloimmune responses via EV signaling constitutes a key knowledge gap.

Based on this background information, we hypothesized that circulating EV proteins might mediate interorgan communication in cardiac allograft rejection. Using an unbiased quantitative proteomic profiling approach in a rat cardiac transplantation model, we sought to map the systemic EV landscape during acute rejection. This analysis revealed a distinct enrichment of hepatic-derived proteins within the circulating EV pool, pointing to a liver-graft signaling axis. Among these liver-enriched candidates, antithrombin III (ATIII; encoded by *Serpinc1*) emerged as a top target for investigation.

ATIII is a hepatocyte-synthesized serpin protease inhibitor (14), best known as an endogenous anticoagulant factor. However, it also possesses potent antiinflammatory and cytoprotective properties independent of anticoagulation. Prior studies have suggested that ATIII might have the function of suppressing leukocyte adhesion and proinflammatory cytokine release (15, 16). We therefore postulated that the observed increase in EV-encapsulated ATIII during rejection might represent an adaptive, compensatory protective response by the liver to counteract rejection-associated inflammation.

In this study, we investigated the functional necessity and therapeutic potential of this hepatic-derived EV signaling pathway. Our integration of lineage tracing with loss- and gain-of-function models demonstrated that hepatocyte-derived EVs mediate this liver-heart axis, and that ATIII acts as a physiological regulator to dampen cardiac allograft rejection. Furthermore, we defined a mechanism wherein this hepatic signal orchestrates protection not through simple anticoagulation, but by mobilizing cytoprotective pathways to reprogram the intragraft immune microenvironment. These findings establish the liver as an active remote regulator of transplant immunity and highlight ATIII-related pathways as a promising therapeutic target for heart transplantation rejection.

## Results

### Characterization of circulating EVs during cardiac allograft rejection.

**Figure 1:**
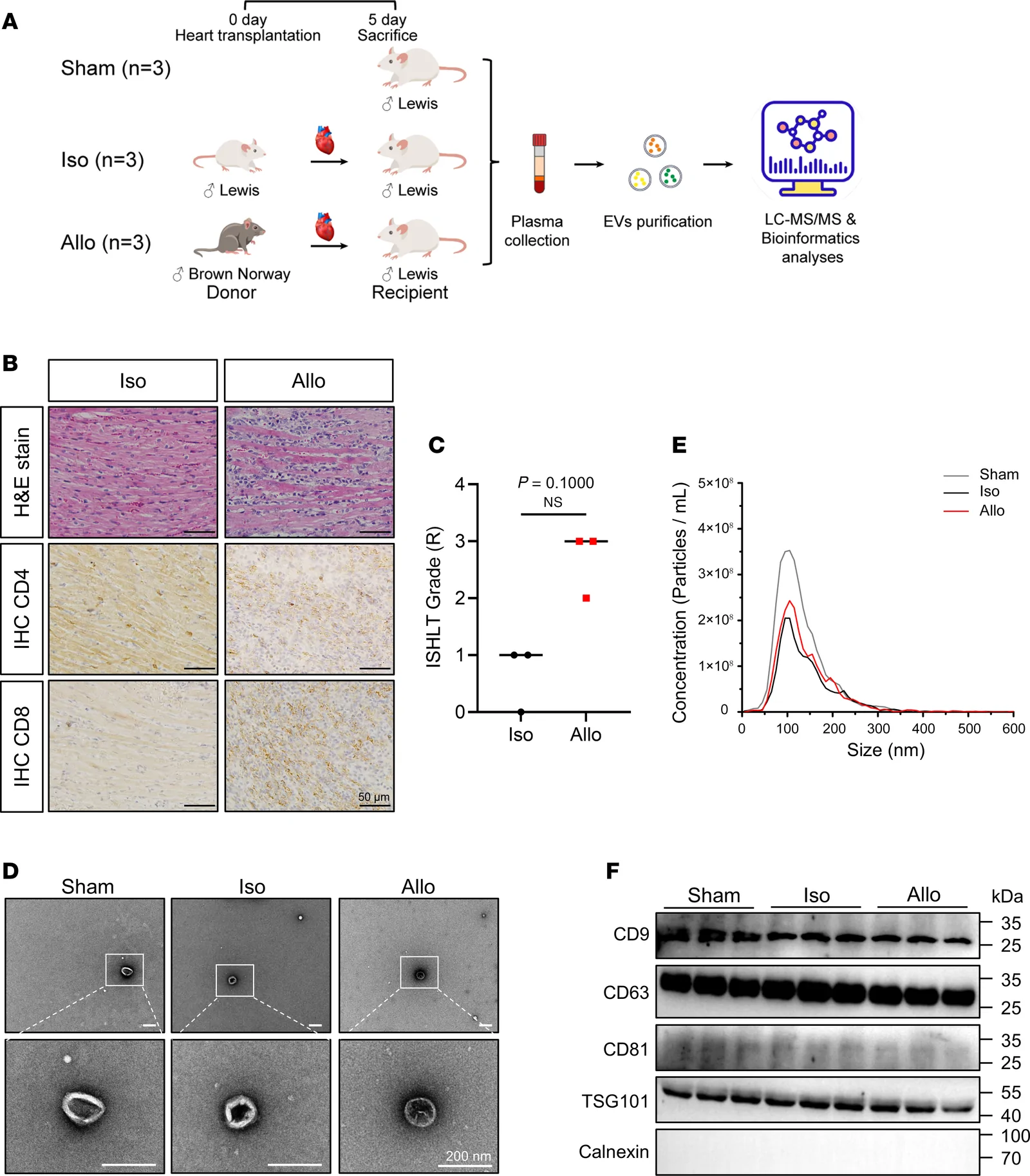
Characterization of plasma-derived EVs in cardiac allograft rejection models. (**A**) Experimental workflow for plasma-derived EV proteomics. Heterotopic heart transplantation was performed in 3 cohorts: the sham-operated controls (Sham), isogeneic group (Iso, Lewis to Lewis), and allogeneic group (Allo, Brown Norway to Lewis). Plasma and graft tissues were collected at postoperative day 5 for proteomic profiling and histopathological assessment. (**B**) Representative H&E, CD4, and CD8 IHC images of cardiac graft sections from 3 rats per group. Scale bar: 50 μm. (**C**) Cellular rejection severity quantified using the 2004 International Society for Heart and Lung Transplantation (ISHLT) guidelines (*n* = 3, Mann-Whitney *U* test). Data are presented as individual values with median. (**D**) Representative transmission electron microscopy images of purified EVs from 3 rats per group. Scale bar: 200 nm. (**E**) Representative nanoparticle tracking analysis size distribution profiles from pooled EV samples. (**F**) Representative Western blots of canonical EV markers (CD9, CD63, CD81, and TSG101) and the cellular contaminant Calnexin (3 rats per group). Quantification of the bands is presented in Supplemental Figure 1. Blots are representative of 2 independent experiments.

To delineate signatures of circulating EVs during acute cardiac allograft rejection, we conducted proteomic profiling of plasma-derived EVs 5 days after transplantation (Figure 1A), concurrent with histological assessment of graft inflammation (Figure 1, B and C). Allogeneic (Allo) but not isogeneic (Iso) grafts exhibited extensive myocardial damage, including cardiomyocyte necrosis, disorganized myocardial fibers, diffuse interstitial leukocyte infiltration, and petechial hemorrhages, confirming that the observed severe injury in allografts was due to immune rejection rather than surgical trauma.

Next, the ultrastructural and molecular features of plasma EVs were validated across groups. Transmission electron microscopy images revealed circular/elliptical double-membrane vesicles (30–150 nm diameter), with high-magnification imaging confirming the canonical cup-shaped morphology (Figure 1D), consistent with the ultrastructure of EVs. Nanoparticle tracking analysis demonstrated that EV yields and size distributions were largely comparable across the sham, Iso, and Allo groups, suggesting that the process of EV biogenesis was not grossly altered by transplant or rejection status (Figure 1E). Isolation consistency was further supported by comparable particle-to-protein ratios across the sham, Iso, and Allo groups (1.51 ± 0.23, 1.02 ± 0.19, and 1.35 ± 0.81 × 10^8^ particles/μg, respectively). Western blot analysis confirmed the presence of canonical EV markers (CD9, CD63, CD81, and TSG101) and the absence of the cellular contaminant Calnexin in all isolates (Figure 1F). Quantitative densitometry revealed stable expression levels of all 4 EV markers across the sham, Iso, and Allo groups, with no statistically significant differences (Supplemental Figure 1; supplemental material available online with this article; https://doi.org/10.1172/jci.insight.200422DS1). Collectively, these data demonstrate the successful purification of EVs with preserved structural integrity, establishing a reliable foundation for subsequent proteomic profiling.

### Proteomic profiling of plasma-derived EVs during cardiac allograft rejection.

**Figure 2:**
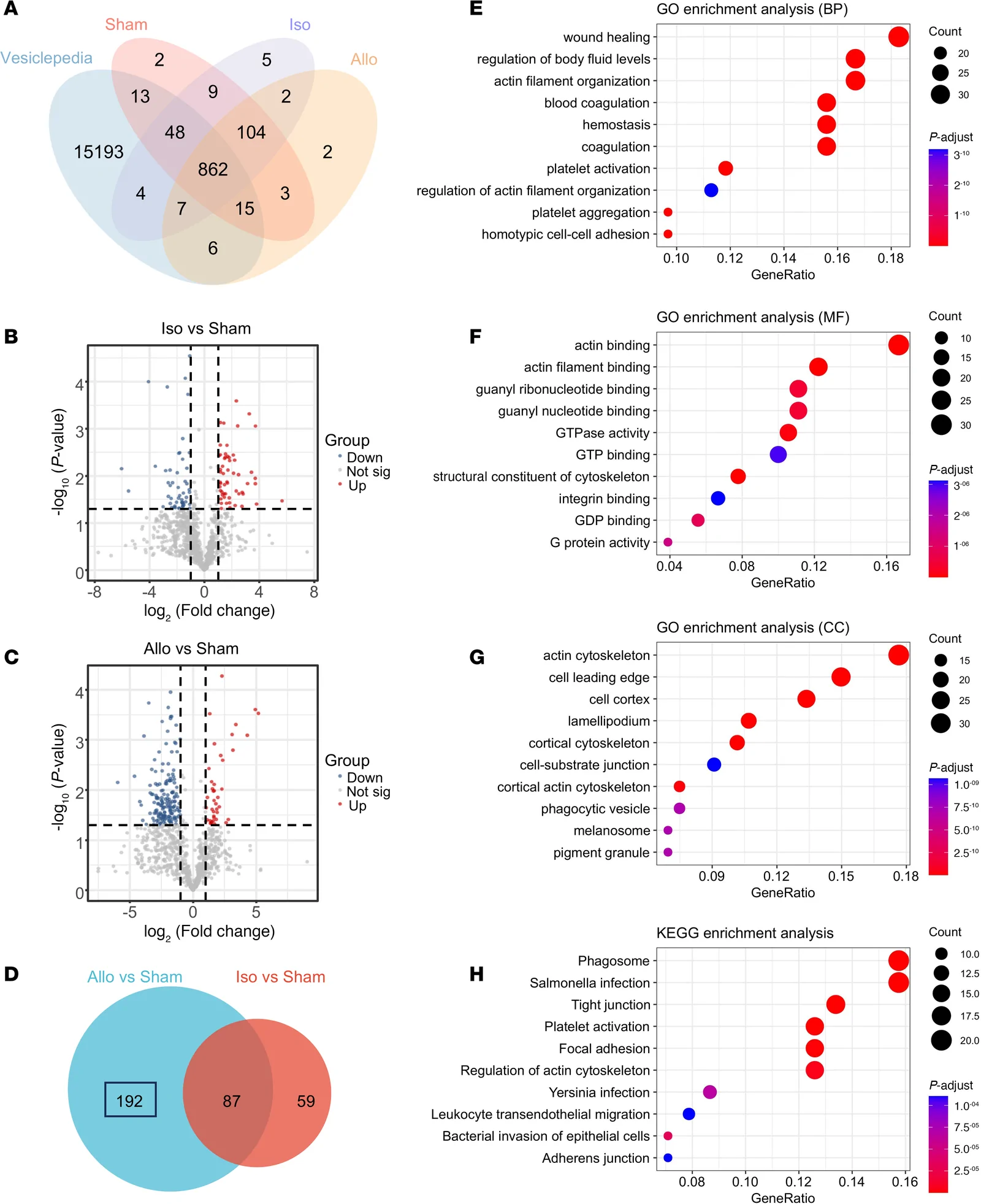
Proteomic profiling of plasma-derived EVs in cardiac allograft rejection. (**A**) Venn diagram comparing proteins identified in the EV samples versus curated EV proteins in the Vesiclepedia database. (**B**) Volcano plots of differentially expressed proteins (DEPs) comparing the Iso group with the sham control. (**C**) Volcano plots of DEPs comparing the Allo group with the sham control. DEPs were identified via the limma package (|log_2_FC| > 1, *P* < 0.05). (**D**) Venn analysis of immune rejection-associated proteins. The boxed region represents 192 acute rejection-related proteins selected for functional annotation. (**E**–**H**) Functional enrichment analysis. Gene Ontology (GO) terms for biological processes (**E**), molecular functions (**F**), cellular components (**G**), and top-enriched Kyoto Encyclopedia of Genes and Genomes (KEGG) pathways (**H**). Proteomic analysis was performed on EVs isolated from 3 rats per group (*n* = 3 biological replicates). Enrichment *P* values were calculated by hypergeometric test and Benjamini-Hochberg–adjusted FDR < 0.05.

Using unbiased liquid chromatography–tandem mass spectrometry (LC-MS/MS) proteomics, we profiled the protein cargo of circulating EVs in each group. The total protein yield was comparable across groups. To validate our EV proteome against established datasets, we cross-referenced identified proteins with the Vesiclepedia database (v5.1, 2023) (17), a publicly available repository of EV proteins. This comparison revealed that approximately 90% of identified proteins across groups aligned with previously reported EV components (sham: 938/1056, 88.8%; Iso: 921/1041, 88.5%; Allo: 890/1001, 88.9%) (Figure 2A). Then, we performed differential expression protein (DEP) analysis to pinpoint proteins altered during rejection. Volcano plots contrasting Iso versus sham (Figure 2B) and Allo versus sham (Figure 2C) revealed numerous protein changes in both transplant conditions relative to controls. By intersecting these 2 DEP sets above, we filtered out the overlap (likely reflecting perioperative confounders, such as anesthesia, surgical trauma, and hemorrhage), and focused on 192 candidate proteins uniquely or more strongly dysregulated in allograft recipients (Figure 2D). These 192 proteins were considered putative acute rejection–associated EV proteins.

Gene Ontology (GO) analysis revealed that immune rejection-associated DEPs identified in EVs were primarily enriched in biological processes related to coagulation, hemostasis, platelet activation, and cytoskeletal remodeling, with molecular functions including actin binding and GTPase activity (Figure 2, E and F). Additionally, cellular component analysis showed that these immune rejection-associated DEPs were highly enriched in vesicles and the extracellular matrix, further confirming efficient EV isolation (Figure 2G). Consistently, Kyoto Encyclopedia of Genes and Genomes (KEGG) pathway analysis indicated involvement of phagosome, platelet activation, and leukocyte transendothelial migration (Figure 2H). These processes converge on the known pathophysiology of acute rejection, which involves inflammation, microvascular injury, and immune cell infiltration. The prominence of coagulation and platelet pathways in the EV proteome is intriguing, given the interplay between clotting cascades and inflammation (often termed “thromboinflammation”) in graft rejection.

### Tissue-origin analysis and in vivo tracing reveal a predominant hepatic contribution to the EV signature of rejection.

**Figure 3:**
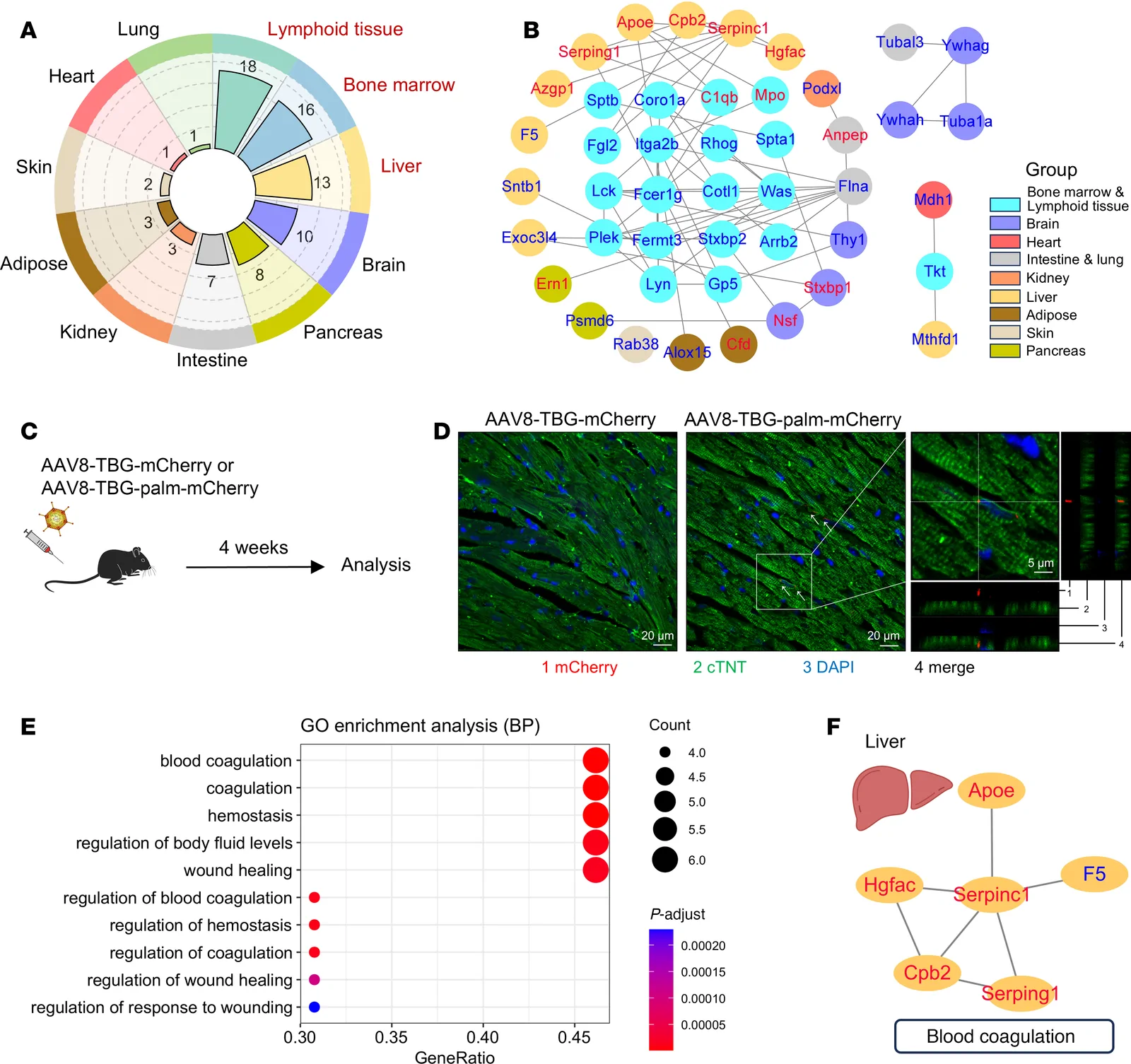
Mapping tissue origins of plasma EV proteins in cardiac allograft rejection. (**A**) Polar bar chart displays the count of tissue-enhanced DEPs identified in allogeneic recipients. (**B**) Protein-protein interaction analysis of tissue-specific proteins altered in allogeneic recipients. Node colors indicate corresponding tissue origins. (**C**) Experimental scheme illustrating the in vivo EV tracing strategy. AAV8-TBG-palm-mCherry, expressing a hepatocyte-specific membrane-localized mCherry fluorescent protein, was administered to mice via tail vein injection. Mice were then maintained for 4 weeks to allow for hepatocyte transduction and subsequent mCherry-labeled EV production and systemic distribution. Cardiac tissues were harvested at the experimental endpoint for confocal analysis. (**D**) Representative confocal images demonstrating mCherry fluorescence in the myocardium of AAV8-TBG-palm-mCherry–injected mice. White arrows indicate liver-derived mCherry-positive EVs distributed within the cardiac parenchyma. The panel on the right provides a high-magnification view (magnified from the boxed area) with an orthogonal projection (*x*-*z* and *y*-*z* planes), confirming the internalization and spatial localization of hepatic EVs within the myocardial tissue. Images are representative of 3 mice per group (*n* = 3). Scale bars: 200 μm (left panel) and 5 μm (right magnified view). (**E**) Functional enrichment in liver. GO biological process (BP) analysis of DEPs enhanced in hepatic tissue. (**F**) Integrated network of liver-enhanced proteins and associated signaling cascades (blood coagulation) dysregulated in allograft recipients. Label colors in **B** and **F** indicate protein expression changes in the Allo group compared with the sham group: blue denotes downregulated proteins, red denotes upregulated proteins. Proteomic data in **A**, **B**, **E**, and **F** represent *n* = 3 independent biological replicates. Enrichment *P* values were calculated by hypergeometric test and Benjamini-Hochberg–adjusted FDR < 0.05.

We next sought to determine the tissue sources of these EV proteins elevated during rejection. Using tissue-enhancement criteria from the Human Protein Atlas, each of the 192 rejection-associated EV proteins was mapped to potential organs of origin (based on where it is most abundantly expressed under normal conditions). This analysis revealed multiorgan involvement in the EV proteome changes. As expected, many proteins originated from immune-related organs: lymphoid tissues (spleen, lymph nodes, thymus) and bone marrow, which contributed a substantial fraction of the altered proteins (Figure 3A and Table 1). These included proteins involved in leukocyte activation and immune signaling (e.g., Lyn, Lck, components of complement, and immunoglobulin receptors) (Supplemental Figure 2), many of which were downregulated in EVs during rejection, possibly reflecting consumption or migration of immune cells during an active response.

Strikingly, among nonimmune organs, the liver stands out as a major source of EV proteins associated with rejection. The liver exhibited the most pronounced enrichment of tissue-specific proteins in the Allo group, surpassing even the contributions from the heart itself (Figure 3, A and B). In fact, only one cardiac-specific protein (malate dehydrogenase 1, Mdh1; ref. 18) was identified as differentially expressed, and it was downregulated in allograft EVs, suggesting that relatively few proteins uniquely originate from the graft heart in this acute phase. Instead, hepatic proteins constituted a significant portion of the EV changes, indicating a systemic response emanating from the liver. Other organs like the brain, pancreas, and intestines also showed some representation, but to a lesser extent than the liver.

To visually track the interorgan transport of hepatic EVs, we established an in vivo reporter system by administering a hepatocyte-specific adeno-associated virus (AAV8-TBG-palm-mCherry) to express membrane-localized mCherry fluorescent protein in the liver (19, 20) (Figure 3C). Four weeks after injection, mCherry signals were exclusively localized to the hepatocyte plasma membranes, confirming both successful AAV-mediated transduction and the efficacy of palmitoylation-directed labeling (Supplemental Figure 3). Subsequent examination of the myocardium revealed discrete mCherry-positive punctate signals scattered within the heart tissue (Figure 3D). Orthogonal projection analysis confirmed that the mCherry-positive puncta were spatially embedded within the tissue architecture rather than being superficial artifacts (Figure 3D). These observations offer direct evidence that liver-derived EVs enter the systemic circulation and physically localize to the heart, establishing a tangible link in the liver-heart signaling axis.

Further characterization of the hepatic EV cargo revealed a specific upregulation of proteins involved in coagulation and complement regulation during rejection (Figure 3, E and F). Unlike the downregulation observed in lymphoid- and bone marrow–derived structural proteins, multiple liver-derived proteins were upregulated in the EVs of rats in organ rejection, particularly those related to coagulation and complement (e.g., *Serpinc1*, *Apoe*, coagulation factors, and complement inhibitors). This pattern suggests that during acute rejection, the liver mounts a response that includes increased EV release of coagulation regulatory proteins, potentially as a counterregulatory mechanism to the thromboinflammatory environment. These results indicate that acute cardiac allograft rejection is accompanied by coordinated multiorgan engagement, as reflected in the circulating EV proteome. The hepatic contribution is especially prominent, aligning with the concept that the liver, as a central metabolic and immunological organ, reacts to and possibly modulates the systemic immune response through the targeted delivery of regulatory EV cargo to the transplanted heart.

### Identification of ATIII as a key liver-derived EV protein during cardiac allograft rejection.

Our proteomic profiling identified the liver as the primary source of dysregulated EV cargo during rejection, positioning hepatic EVs as critical signal carriers. To systematically identify the most pivotal effector molecule from this liver-enriched EV proteome, we employed a multi-step bioinformatic prioritization strategy.

**Figure 4:**
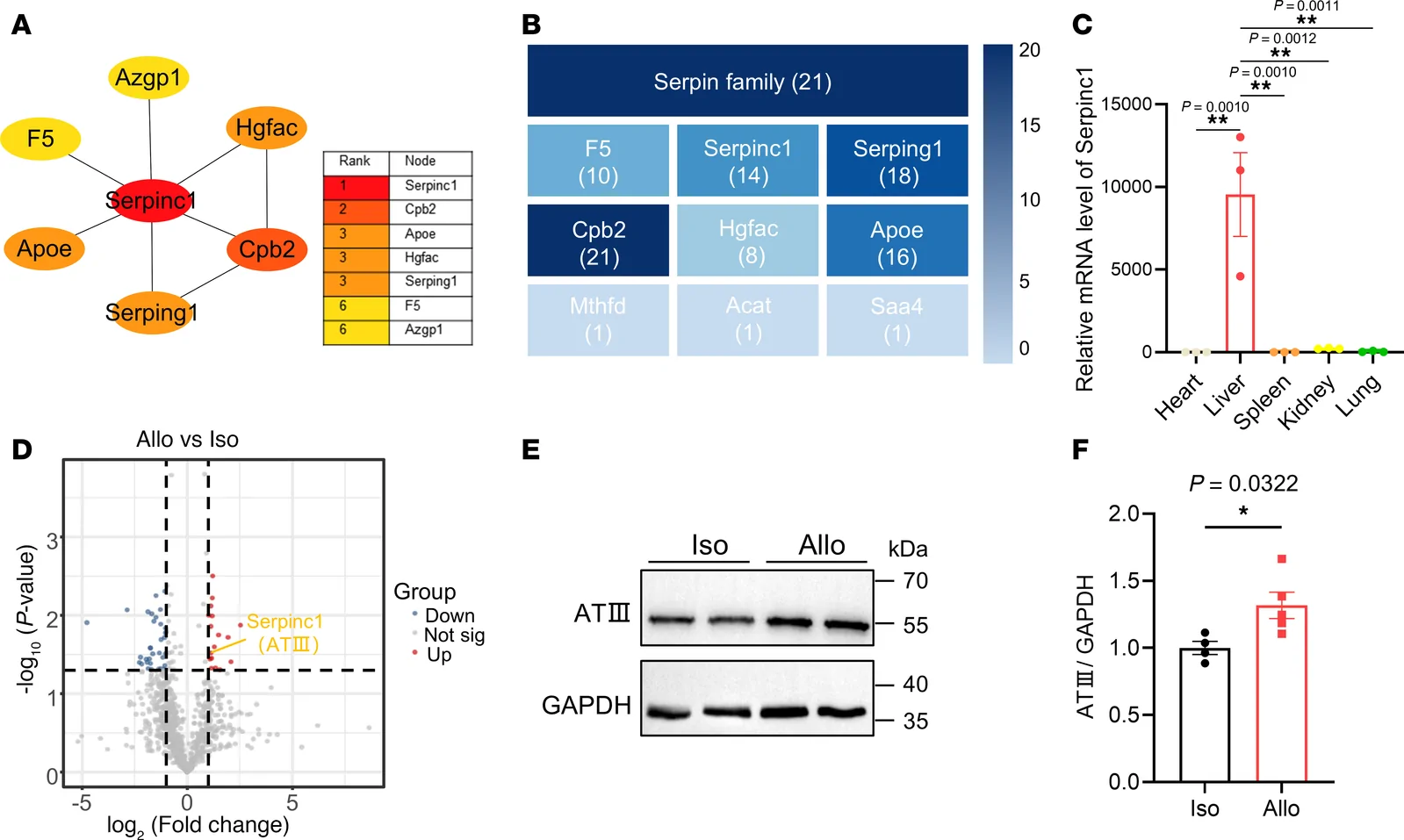
Hepatic EV protein signatures in cardiac allograft rejection. (**A**) Protein-protein interaction analysis of liver-enhanced DEPs in allograft recipients. Node colors reflect topological ranking (dark red = highest connectivity). Hub genes were identified using the maximal clique centrality algorithm via CytoHubba. (**B**) Word cloud visualization of high-frequency hepatic tissue-enhanced proteins identified from the top 25 GO biological processes for DEPs. Module colors correspond to term occurrence frequency. (**C**) RT-qPCR analysis of *Serpinc1* mRNA levels in the indicated tissues from Lewis rats (*n* = 3 biological replicates). (**D**) Volcano plot showing DEPs in plasma EVs from the Allo group versus the Iso group. (**E**) Representative Western blot analysis of antithrombin III (ATIII) expression in recipient livers. Blots are representative of 2 independent experiments. (**F**) Densitometric quantification of ATIII protein levels (Iso group, *n* = 4; Allo group, *n* = 5 biological replicates). Data presented as mean ± SEM. Statistical significance in **C** was determined by 1-way ANOVA followed by Tukey’s multiple-comparison test. For **F**, statistical significance was determined by an unpaired Student’s *t* test. *P < 0.05, **P < 0.01.

A protein-protein interaction network was first constructed for all liver-derived DEPs. Within this network, the CytoHubba algorithm identified *Serpinc1* (encoding ATIII) as a top-ranked hub gene (Figure 4A), reflecting its central role in the molecular interaction landscape. This topological prominence was reinforced by quantitative abundance; indeed, ATIII emerged as one of the most substantially upregulated candidates, ranking among the top 4 liver-derived proteins by fold change (Supplemental Table 1). To assess the biological significance of this upregulation, we conducted a word-frequency analysis of the enriched pathways (shown in Figure 3E). This analysis highlighted a predominant focus on serpin-mediated biology, with 21 of the top 25 enriched pathways directly associated with the serpin family. Notably, *Serpinc1* served as a core constituent in approximately two-thirds (14 of 21) of these serpin-related processes, which spanned critical functions including coagulation, hemostasis, and inflammatory modulation (Figure 3E and Figure 4B). These analyses position ATIII not merely as an upregulated protein, but as a functional nexus characterized by both topological centrality and high quantitative induction. Given that ATIII modulates vascular integrity and inflammation beyond its canonical anticoagulant functions (15, 16) and considering the established efficacy of recombinant ATIII in transplantation (21), we prioritized it to explore the liver-heart axis.

To validate these findings, we performed reverse transcription–quantitative real-time PCR (RT-qPCR), which confirmed greater than 1,000-fold higher *Serpinc1* mRNA in Lewis rat liver versus heart, kidney, spleen, and lung (Figure 4C), aligning with its hepatocyte-specific synthesis. Proteomic profiling of plasma EVs revealed a significant upregulation of ATIII in the Allo group compared with both the Iso and sham control groups (fold change = 2.20 and 2.86, respectively; *P* < 0.05). The lack of significant difference between the Iso and sham groups suggests that ATIII upregulation is a specific response to alloimmune-mediated rejection rather than a nonspecific effect of surgical trauma (Figure 4D and Supplemental Table 1). Consistent with these findings, Western blot analysis further confirmed elevated ATIII protein expression in the livers of allograft recipients (Figure 4, E and F).

To test whether ATIII is a genuine luminal cargo of liver-derived EVs, rather than a co-isolated soluble contaminant, we performed a proteinase K (PK) protection assay to determine its subvesicular localization. As shown in Supplemental Figure 4, ATIII protein remained intact and resistant to PK digestion under membrane-intact conditions, whereas membrane-anchored CD9 was degraded. Upon disruption of the lipid bilayer with Triton X-100, ATIII became susceptible to proteolysis. These biochemical findings confirm that ATIII is carried within EVs rather than as a soluble contaminant. Given ATIII’s dual role in anticoagulation and inflammation control, we hypothesized that its upregulation might be an attempt by the host to counterbalance the prothrombotic and proinflammatory state of acute rejection.

### Depletion of endogenous circulating EVs or specific silencing of hepatic ATIII exacerbates inflammatory responses and accelerates rejection.

**Figure 5:**
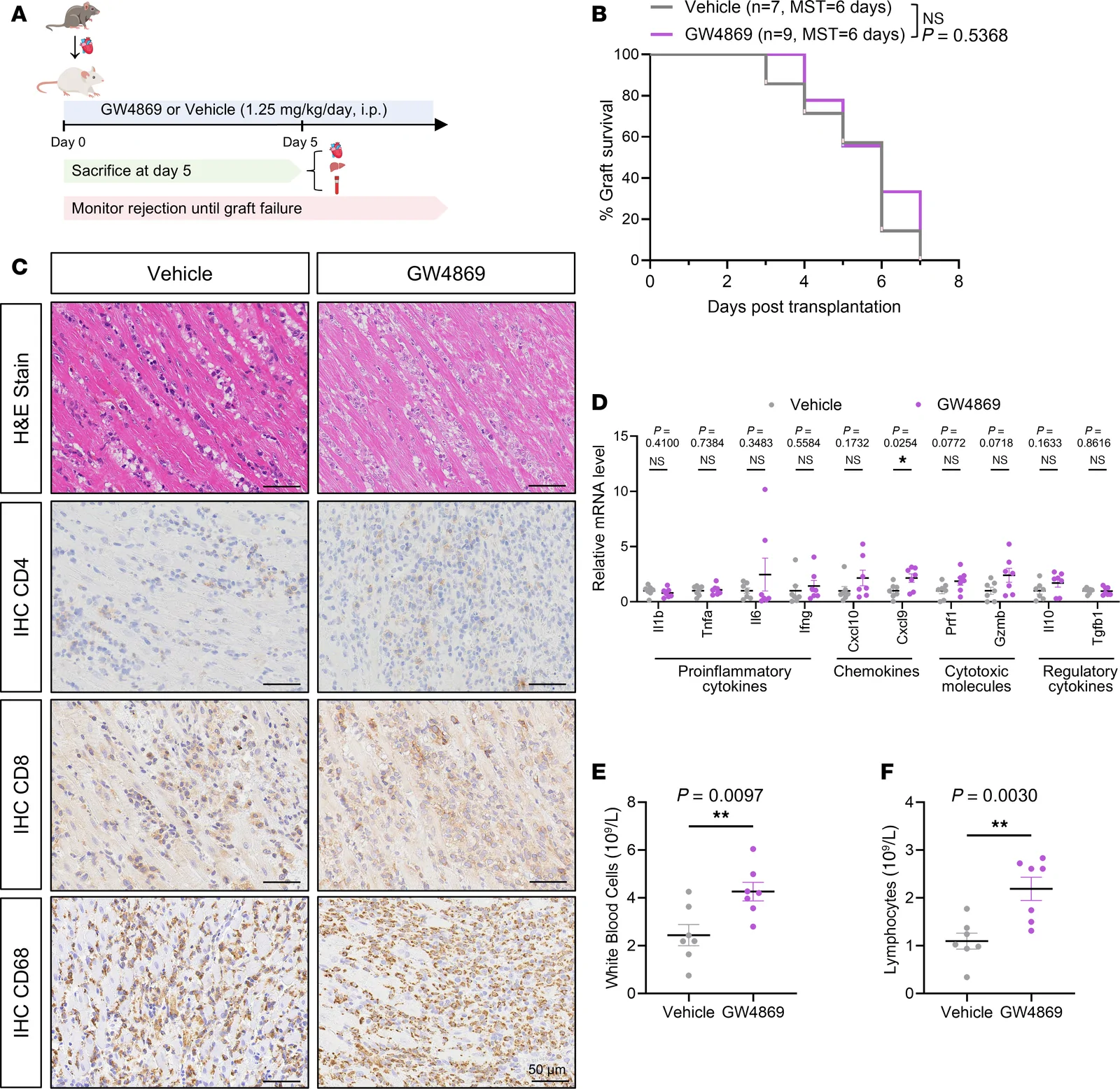
Depletion of endogenous circulating EVs exacerbates systemic and intragraft inflammatory responses. (**A**) Experimental workflow. Lewis recipients were treated with GW4869 (1.25 mg/kg, i.p.) or vehicle daily, starting 1 hour before Brown Norway heart transplantation. Grafts were monitored for survival or harvested at postoperative day 5 for mechanistic analysis. (**B**) Kaplan-Meier curves comparing allograft survival between groups. Log-rank test (vehicle, *n* = 7; GW4869, *n* = 9). (**C**) Histological and IHC assessment. Representative H&E, CD4, CD8, and CD68 IHC staining of cardiac allografts from the biological replicates. Scale bar: 50 μm. (**D**) Graft inflammatory profile. Relative mRNA levels of proinflammatory cytokines (*Il1b, Tnfa, Il6, Ifng*), chemokines (*Cxcl9, Cxcl10*), cytotoxic molecules (*Prf1, Gzmb*), and regulatory factors (*Tgfb1, Il10*) in cardiac allografts. (**E** and **F**) Systemic immune monitoring. Peripheral WBC counts (**E**) and lymphocyte counts (**F**) in recipient rats. Data are presented as mean ± SEM. Sample sizes for **C**–**F**: vehicle, *n* = 7; GW4869, *n* = 7 biological replicates. Unpaired 2-tailed Student’s *t* test determined statistical significance for comparisons in **D**–**F**. *P < 0.05, **P < 0.01, ns, not significant. MST, median survival time.

To evaluate whether basal levels of circulating EVs function as a physiological regulator of the alloimmune response, we administered GW4869, a specific inhibitor of neutral sphingomyelinase-2 (nSMase2) known to suppress EV biogenesis (19, 21), to recipient rats (Figure 5A). GW4869 treatment did not significantly shorten graft survival compared with vehicle controls, and both groups succumbed to rejection by postoperative day 6 (Figure 5B). This lack of statistical difference is likely attributable to the rapid kinetics and high severity of acute rejection inherent to this fully MHC-mismatched model, which limits the potential to observe further acceleration of graft loss. Histological assessment supported this finding, as morphological damage appeared to have reached a peak severity in both cohorts. H&E staining of cardiac allografts displayed comparably severe acute rejection, characterized by widespread myocyte necrosis and massive infiltration of mononuclear inflammatory cells (Figure 5C). Similarly, IHC demonstrated robust infiltration of CD4^+^ T cells, CD8^+^ T cells, and CD68^+^ macrophages (Figure 5C).

Despite these similar histological profiles, molecular analysis of key inflammatory and cytotoxic mediators revealed an underlying hyperinflammatory signature in EV-depleted recipients. RT-qPCR showed a significant upregulation of *Cxcl9*, a key chemokine for T cell recruitment (Figure 5D). This loss of immunoregulation extended beyond the local graft environment into the systemic circulation. Inhibiting EV secretion resulted in a marked expansion of the circulating leukocyte population, characterized by a significant increase in both total WBC counts and the lymphocyte pool (Figure 5, E and F, and Supplemental Table 2). These hematological shifts, coupled with the intragraft molecular changes, reinforce the role of basal circulating EVs as a critical systemic brake on inflammatory activation.

**Figure 6:**
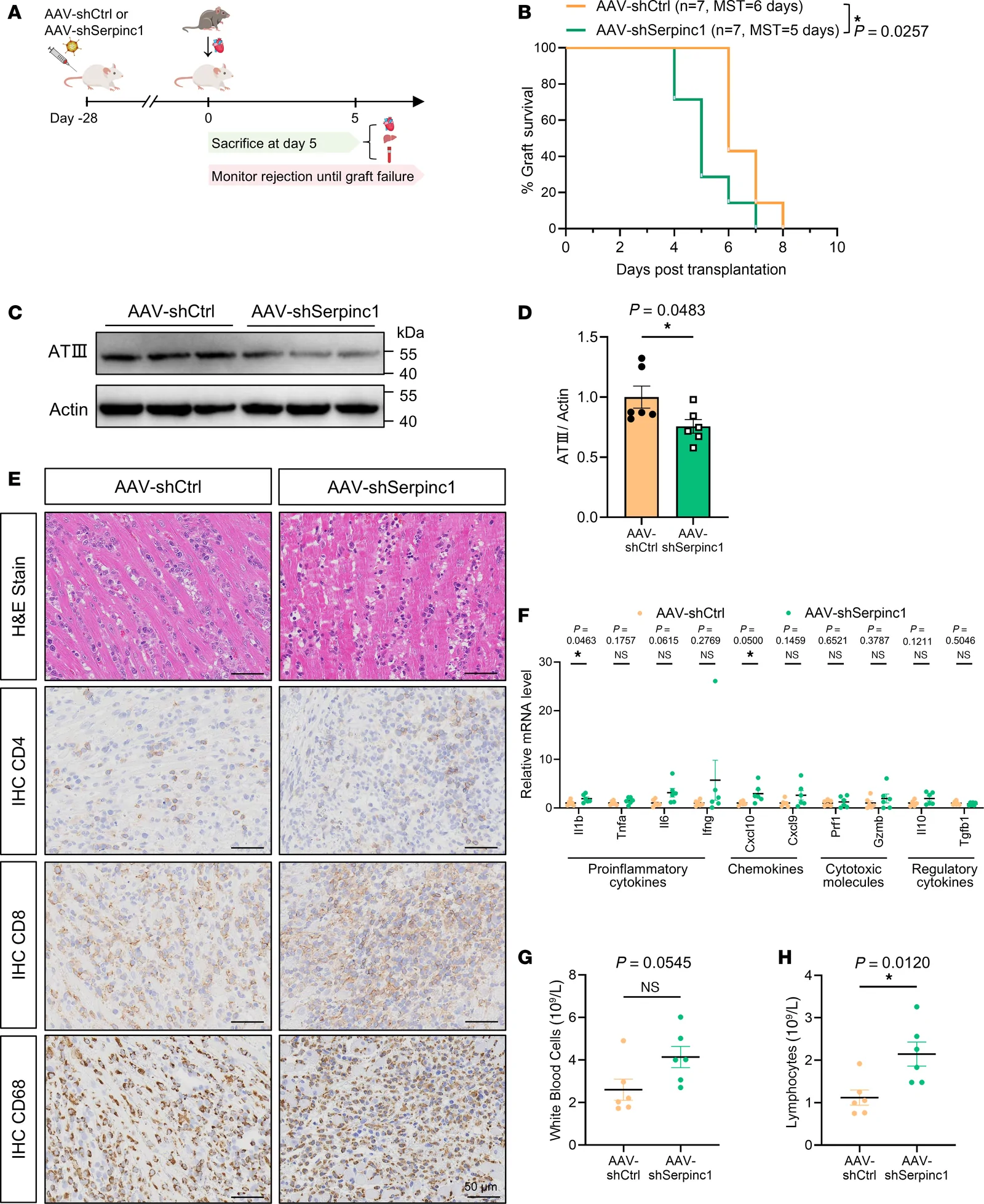
Genetic silencing of hepatic ATIII accelerates cardiac allograft rejection and intensifies immune infiltration. (**A**) Experimental workflow. Lewis rats were i.v. injected with AAV-shSerpinc1 or AAV-shCtrl to induce hepatocyte-specific ATIII knockdown 4 weeks before transplantation. Endpoints included graft survival and mechanistic assessment at postoperative day 5. (**B**) Kaplan-Meier curves comparing allograft survival between groups (log-rank test; shCtrl, *n* = 7; shSerpinc1, *n* = 7). (**C** and **D**) Hepatic ATIII knockdown verified by representative Western blots (**C**, 3 rats per group shown) and quantitative analysis (**D**, *n* = 6 biological replicates). Blots are representative of 2 independent experiments. (**E**) Histological and IHC assessment. Representative H&E, CD4, CD8, and CD68 IHC staining of cardiac allografts from the biological replicates. Scale bar: 50 μm. (**F**) Graft inflammatory profile. Relative mRNA levels of proinflammatory cytokines (*Il1b*, *Tnfa*, *Il6*, *Ifng*), chemokines (*Cxcl9*, *Cxcl10*), cytotoxic molecules (*Prf1*, *Gzmb*), and regulatory factors (*Tgfb1*, *Il10*) in cardiac allografts. (**G** and **H**) Systemic immune monitoring. Peripheral WBC counts (**G**) and lymphocyte counts (**H**) in recipient rats. Data are presented as mean ± SEM. Sample sizes for **C**–**H**: shCtrl, *n* = 6; shSerpinc1, *n* = 6 biological replicates. Statistical significance in **F** for *Il6* and *Cxcl10* was determined by 2-tailed Welch’s *t* test due to unequal variance; all other comparisons in **D** and **F**–**H** were analyzed by unpaired 2-tailed Student’s *t* test. *P < 0.05, ns, not significant.

To pinpoint whether the liver-derived ATIII cargo specifically accounts for this protective phenomenon, we generated a liver-specific knockdown model using an AAV8 vector encoding a shRNA targeting *Serpinc1* (AAV-shSerpinc1) (Figure 6A). Broad EV depletion had minimal effect on graft survival, whereas specific silencing of hepatic ATIII resulted in a measurable acceleration of rejection. Survival analysis revealed that ATIII knockdown significantly shortened allograft survival, with a median survival time of 5 days compared with 6 days in scramble controls (Figure 6B). Western blot analysis confirmed the efficient reduction of ATIII expression in the liver (Figure 6, C and D), which subsequently led to a marked decrease in ATIII cargo within circulating EVs (Supplemental Figure 5A), validating the link between hepatic synthesis and EV content.

Histological and molecular assessments further characterized this accelerated rejection. Histology corroborated graft failure, as morphological damage peaked in both groups. H&E staining of cardiac allografts displayed comparably fulminant acute rejection (Figure 6E). Similarly, IHC confirmed a massive immune attack, with extensive infiltration of CD4^+^ T cells, CD8^+^ T cells, and CD68^+^ macrophages throughout the myocardium in both the knockdown and control cohorts (Figure 6E). However, molecular profiling unveiled an underlying hyperinflammatory signature driving this accelerated failure. RT-qPCR analysis demonstrated a significant upregulation of the proinflammatory cytokine *Il1b* and the T cell chemoattractant *Cxcl10* in the ATIII-knockdown grafts compared with controls (Figure 6F). This molecular intensification indicates that the loss of hepatic ATIII creates a more aggressive cytokine microenvironment.

Beyond the local graft environment, hepatic ATIII loss recapitulated the systemic immune dysregulation observed with broad EV blockade. ATIII knockdown induced a significant expansion of the lymphoid lineage, with absolute lymphocyte counts nearly doubling compared with scramble controls (Figure 6, G and H, and Supplemental Table 4). Importantly, this immune activation in both the GW4869 and ATIII-knockdown cohorts occurred without systemic toxicity or consumptive coagulopathy. Serum biochemistry confirmed that liver (alanine aminotransferase and aspartate aminotransferase) and kidney (creatinine, urea nitrogen) functions, as well as platelet counts, remained comparable between groups (Supplemental Tables 3 and 5). These findings provide functional evidence that the hepatic ATIII system acts as a critical immunomodulatory axis, essential for dampening systemic lymphocyte expansion and maintaining graft homeostasis.

### Hepatocyte-specific ATIII overexpression attenuates cardiac allograft rejection without inducing systemic toxicity.

**Figure 7:**
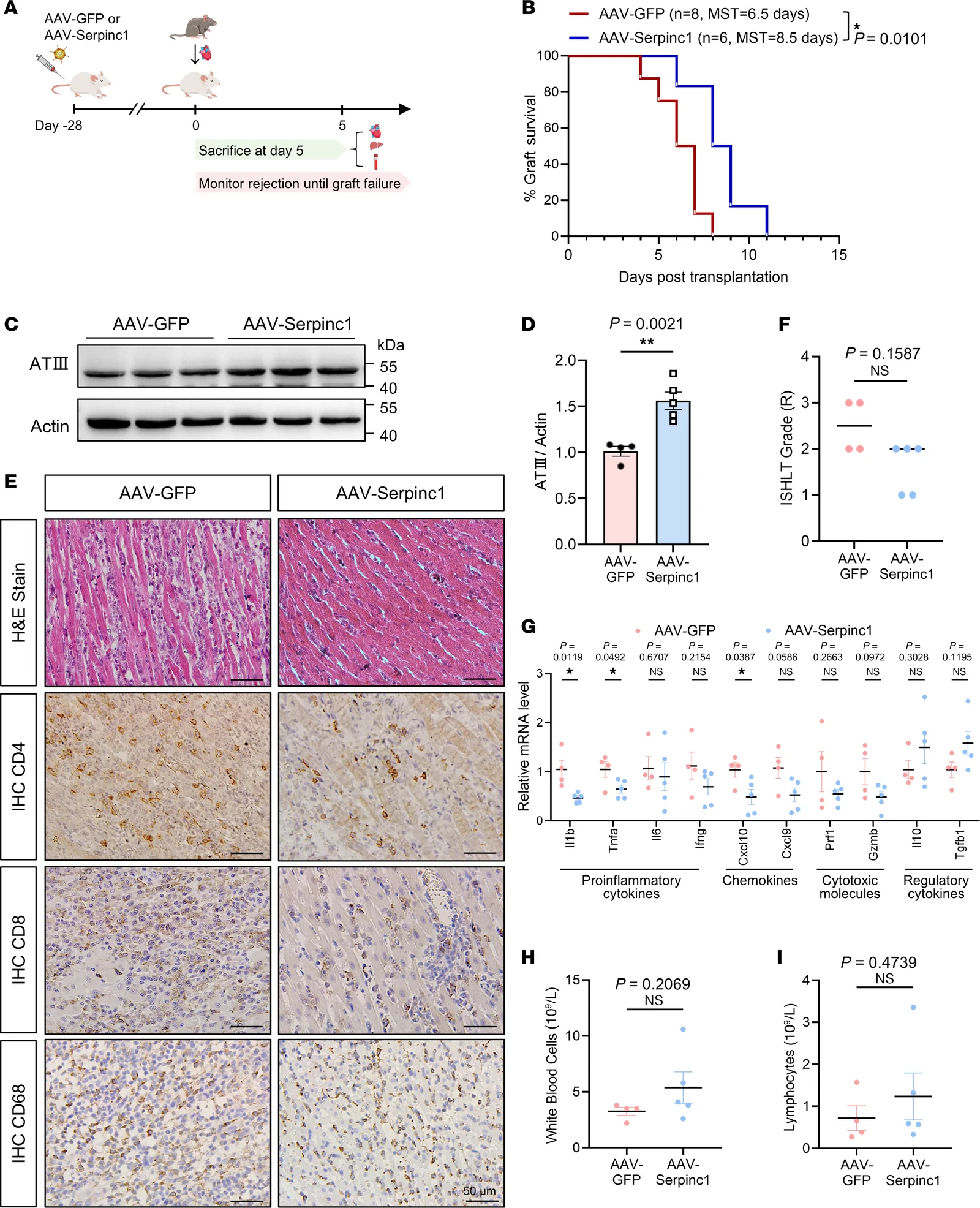
Liver-targeted ATIII overexpression attenuates cardiac allograft rejection. (**A**) Experimental workflow. Lewis rats were i.v. injected with AAV-Serpinc1 or AAV-GFP (control) for hepatocyte-specific overexpression prior to Brown Norway heart transplantation. For survival analysis, graft failure was determined by the loss of palpable cardiac contractions. For mechanistic studies, heart allografts, liver, and plasma were harvested at postoperative day 5. (**B**) Kaplan-Meier curves comparing allograft survival between groups. Log-rank test (AAV-GFP, *n* = 8; AAV-Serpinc1, *n* = 6). (**C** and **D**) Hepatic ATIII verification. Representative Western blots (**C**) and quantitative analysis (**D**) of ATIII expression in recipient livers. Blots are representative of 2 independent experiments. (**E**) Representative H&E, CD4, CD8, and CD68 IHC staining of cardiac allografts from the biological replicates. Scale bar: 50 μm. (**F**) International Society for Heart and Lung Transplantation (ISHLT) grading based on allograft histology. (**G**) Graft inflammatory profile. Relative mRNA levels of proinflammatory cytokines (*Il1b, Tnfa, Il6, Ifng*), chemokines (*Cxcl9, Cxcl10*), cytotoxic molecules (*Prf1, Gzmb*), and regulatory factors (*Tgfb1, Il10*) in cardiac allografts from control and hepatocyte-specific ATIII-overexpressing recipients. (**H** and **I**) Systemic immune monitoring. Peripheral WBC counts (**H**) and lymphocyte counts (**I**) in recipient rats. Data are presented as individual values with median for **F**, and as mean ± SEM for other bar graphs (**D** and **G**–**I**). Sample sizes for **C**–**I**: AAV-GFP, *n* = 4; AAV-Serpinc1, *n* = 5 biological replicates. Statistical significance was determined by Mann-Whitney *U* test for **F**, 2-tailed Welch’s *t* test for *Il1b* in **G** and **H**, unpaired 2-tailed Student’s *t* test for all other comparisons in **D**, **G**, and **I**. *P < 0.05, **P < 0.01, ns, not significant.

Having established the necessity of the hepatic ATIII in mediating the liver-heart axis, we next investigated the therapeutic potential of its enhancement. We employed an AAV-Serpinc1 vector to induce targeted hepatic ATIII overexpression (Figure 7A). Survival analysis demonstrated that boosting this axis significantly prolonged cardiac allograft survival to a median of 8.5 days compared with 6.5 days in the GFP group (Figure 7B). Although the protection was partial in this high-severity, fully MHC-mismatched model, it represents a tangible therapeutic benefit derived from liver-targeted intervention.

This survival benefit was underpinned by molecular and histopathological improvements. Western blot analysis verified the marked upregulation of ATIII within the liver (Figure 7, C and D), which facilitated a corresponding enrichment of ATIII cargo in circulating EVs (Supplemental Figure 5B). H&E staining revealed a marked protective effect. Control allografts (AAV-GFP) exhibited characteristic features of moderate-to-severe acute rejection, including extensive interstitial mononuclear cell infiltration and multifocal myocyte necrosis (Figure 7E). In contrast, grafts from ATIII-overexpressing recipients showed markedly attenuated rejection pathology, characterized by preserved myocardial architecture with only focal, mild inflammatory infiltrates. Quantitative scoring showed a numerically lower median score in the ATIII group, but the difference was not statistically significant (Figure 7F). IHC characterization further corroborated these findings. Control grafts were heavily infiltrated by CD4^+^ T cells, CD8^+^ T cells, and CD68^+^ macrophages, whereas ATIII-overexpressing recipients displayed a marked reduction in the recruitment of these effector immune cells (Figure 7E).

To further define the intragraft molecular landscape, we profiled the expression of the same set of inflammatory and cytotoxic mediators analyzed in our earlier depletion studies. Consistent with the attenuated leukocyte infiltration, ATIII overexpression reversed the hyperinflammatory signature observed in the knockdown and GW4869 groups, leading to a downregulation of proinflammatory genes, including *Il1b*, *Tnfa*, and *Cxcl10* (Figure 7G). Additionally, mRNA levels of cytotoxic effectors *Prf1* (perforin) and *Gzmb* showed a consistent downward trend, with *Gzmb* exhibiting marginal significance (*P* = 0.0972, Figure 7G). This pattern suggests that ATIII overexpression skews the local immune milieu toward a less inflammatory state.

Importantly, this therapeutic efficacy was achieved safely and specifically. Comprehensive safety assessments demonstrated that the treatment was well-tolerated. Histological examination of liver sections from AAV8-treated rats revealed preserved hepatic architecture with intact lobular structures and no signs of necrosis or inflammatory infiltration, confirming that high-level ATIII expression did not induce local tissue damage (Supplemental Figure 6). Systemically, hematological analyses showed that leukocyte counts, lymphocyte subsets, and erythrocyte parameters remained stable after transduction (Figure 7, H and I, and Supplemental Table 6). Furthermore, biochemical profiling indicated that liver and renal function indices were maintained within physiological ranges. Although a mild elevation in amylase and minor fluctuations in phosphorus levels were observed in the AAV-Serpinc1 group, these values remained within clinically acceptable thresholds and were not accompanied by any overt signs of illness (Supplemental Table 7). These data confirm that AAV8-mediated hepatocyte-specific ATIII overexpression is both effective and safe.

We next investigated whether the protective effects of sustained hepatic ATIII overexpression could be recapitulated by the adoptive transfer of exogenous ATIII-enriched EVs. EVs were isolated from ATIII-overexpressing rats (ATIII-EVs) or control rats and injected into allograft recipients. Compared with control EV treatment, ATIII-EV administration did not improve cardiac allograft survival, nor did it significantly alter peripheral leukocyte counts or intragraft inflammatory gene expression (Supplemental Figure 7 and Supplemental Table 8). These results indicate that the therapeutic benefit requires the continuous, physiologically integrated EV production enabled by our AAV8-based strategy, not simple exogenous EV transfer.

### Hepatic ATIII regulates local heme oxygenase-1 expression and immunomodulation beyond canonical anticoagulation.

To elucidate the upstream mechanism underlying the observed immunomodulation, we investigated whether the protective effects of ATIII were driven primarily by its canonical anticoagulant function or by regulating cytoprotective signaling pathways.

**Figure 8:**
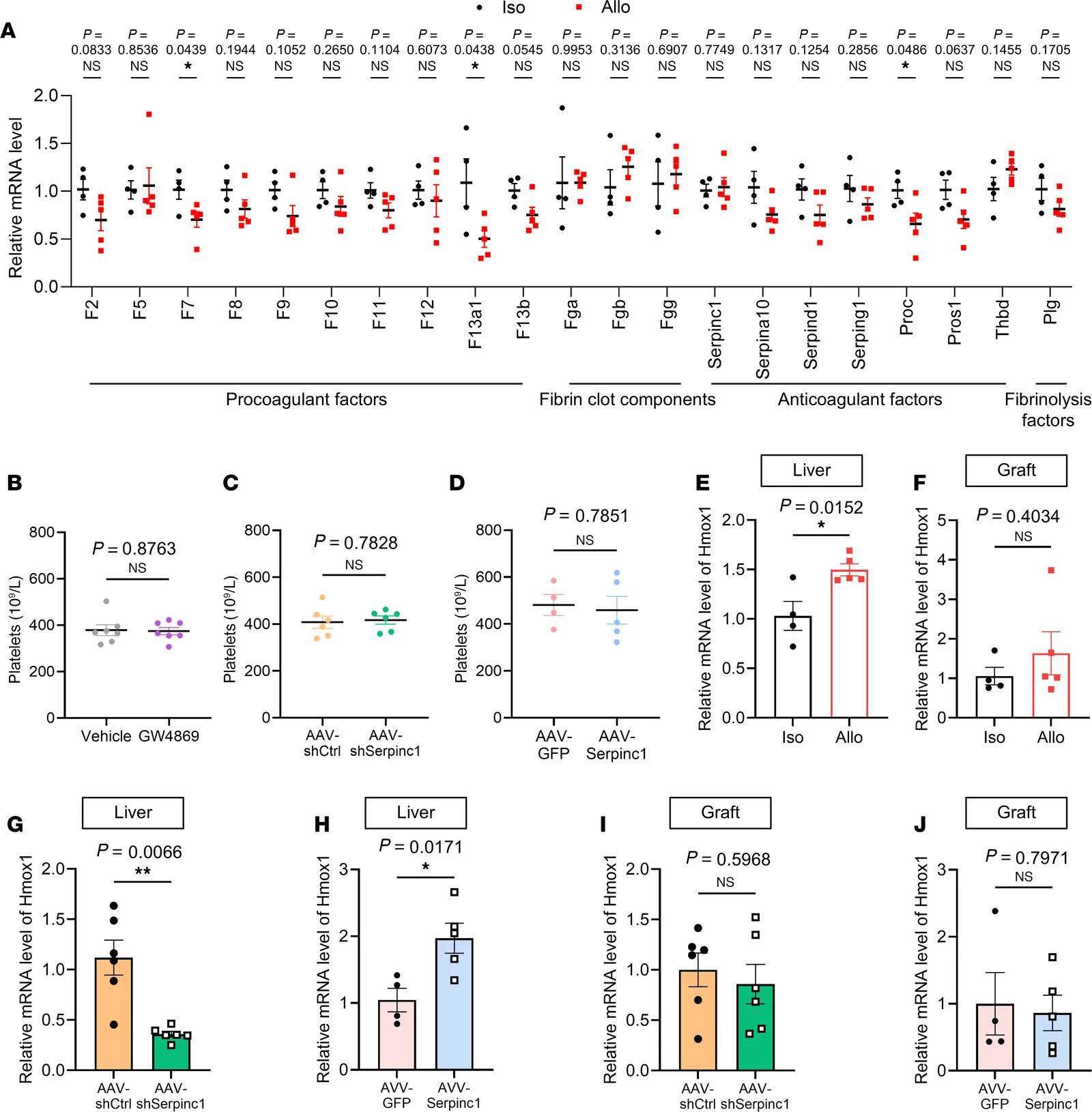
Evaluation of coagulation parameters and regulation of the heme oxygenase-1 pathway. (**A**) Relative mRNA levels of hepatic coagulation factors in Iso and Allo recipients. (**B**–**D**) Platelet counts in recipients after GW4869 treatment (**B**), AAV-mediated Serpinc1 knockdown (**C**), and AAV-mediated Serpinc1 overexpression (**D**) compared with their respective controls. (**E** and **F**) Endogenous Hmox1 response. Relative mRNA levels of *Hmox1* in the liver (**E**) and cardiac allografts (**F**) of Allo recipients versus Iso controls. (**G**–**J**) Regulation of Hmox1 by ATIII. Relative Hmox1 mRNA levels in the liver (**G** and **H**) and cardiac allografts (**I** and **J**) after AAV-mediated Serpinc1 knockdown or overexpression. Data are presented as mean ± SEM. Sample sizes for **A**, **E**, and **F**: Iso, *n* = 4; Allo, *n* = 5. For **B**, vehicle/GW4869, *n* = 7. For **C**, **G**, and **I**, AAV-shCtrl/shSerpinc1, *n* = 6. For **D**, **H**, and **J**, AAV-GFP, *n* = 4; AAV-Serpinc1, *n* = 5. Statistical significance in **G** was determined by 2-tailed Welch’s *t* test; all other comparisons were analyzed by unpaired 2-tailed Student’s *t* test. *P < 0.05, **P < 0.01, ns, not significant.

We first determined whether the transplant rejection or our interventions induced a consumptive coagulopathic state. Analysis of hepatic mRNA levels revealed that the expression of key coagulation factors (including fibrinogen, prothrombin, factor VII, and factor XIIIa) was largely comparable between the Iso and Allo recipients, with only minor variations in select factors (Figure 8A). This indicates that the acute rejection process in this timeframe is not dominated by a liver-driven failure of coagulation factor synthesis.

Furthermore, we assessed systemic coagulation status across all functional intervention groups. Platelet counts, a sensitive indicator of consumptive coagulopathy and thrombotic microangiopathy, remained stable and showed no statistical differences across the GW4869, ATIII-knockdown, or ATIII-overexpression cohorts compared with their respective controls (Figure 8, B–D). Although ATIII intrinsically modulates the coagulation cascade, the stability of platelet counts suggests that the observed acceleration or attenuation of rejection is not a consequence of overt platelet consumption or systemic thrombotic failure. This implies that the therapeutic benefit involves mechanisms extending beyond simple anticoagulation.

To identify the specific immunomodulatory pathway responsible for this protection, we focused on heme oxygenase-1 (HO-1, encoded by *Hmox1*). This focus was informed by our previous observation that HO-1 upregulation substantially prolongs graft survival via innate immune suppression (22). Given that the liver serves as a primary physiological reservoir for HO-1 and that ATIII exerts potent non-anticoagulant antiinflammatory effects (15, 16), we hypothesized that ATIII might confer cardioprotection by mobilizing this hepatic cytoprotective machinery.

To test this hypothesis, we first mapped the endogenous expression profile of *Hmox1* in both the liver and the cardiac allograft during acute rejection. In the liver, *Hmox1* mRNA levels were significantly upregulated in the Allo recipients compared with Iso controls (Figure 8E), suggesting that the liver mounts a vigorous adaptive stress response to the alloimmune challenge. In contrast, analysis of the cardiac tissue revealed no significant difference in *Hmox1* expression between Allo and Iso grafts (Figure 8F). This spatial dissociation suggests that the HO-1 response during rejection is primarily hepatocentric rather than a ubiquitous systemic induction.

We next investigated whether this hepatic HO-1 response is functionally regulated by ATIII. Specific knockdown of hepatic ATIII significantly suppressed *Hmox1* expression in the liver (Figure 8G). Conversely, AAV-mediated overexpression further potentiated hepatic *Hmox1* levels beyond the baseline elevation seen in rejection (Figure 8H). Notably, neither intervention altered *Hmox1* expression within the cardiac allografts (Figure 8, I and J), identifying ATIII as a specific upstream regulator of hepatic, but not myocardial, HO-1. The protective efficacy of the liver-heart axis appears to be mediated by this liver-specific induction of cytoprotective pathways, which subsequently creates a systemic antiinflammatory milieu, rather than being solely attributable to its anticoagulant properties.

## Discussion

Heart transplant rejection remains a complex process influenced by systemic immune networks beyond the graft itself. EV-mediated communication has emerged as a fundamental mechanism of interorgan crosstalk, but its specific role and functional constituents in transplant immunology remain largely unexplored. Here, we reveal that hepatocyte-derived EVs constitute a signaling axis that directly communicates with the distant cardiac allograft to regulate rejection, and we implicate the protein ATIII as a contributor to this immunomodulatory axis.

Our results align with a growing recognition that transplant outcomes are not determined solely by events in the graft and lymphoid organs, but also by systemic interorgan communication. The liver, in particular, is increasingly seen as a central immunometabolic “hub” that can modulate immune responses throughout the body. The concept of the liver as a master regulator of immunity is supported by its unique dual blood supply and role in filtering antigens, as well as its ability to secrete a plethora of immune-active molecules (complement factors, acute phase proteins, cytokine-like hormones, and EVs) (23–25). In the context of transplantation, recent studies underscore that the liver can either exacerbate or alleviate alloimmune reactions. Liver function critically influences transplant immunology, with clinical studies confirming that hepatic dysfunction fundamentally disrupts transplantation pharmacology. This impairment markedly reduces clearance of mTOR antagonists such as everolimus, consequently escalating rejection risk through immunosuppression (26). The liver’s regulatory dominion over rejection pathology is further evidenced by Zhang et al.’s demonstration of PCSK9/CD36-driven macrophage reprogramming during heart transplantation, where hepatic PCSK9 depletion resulted in extended allograft survival and reduced infiltration of inflammatory cells within the cardiac grafts, as well as diminished expansion of alloreactive T cells in the spleens (4). Similarly, Cao et al. revealed that hepatobiliary coagulation factor XI (FXI) paradoxically protects against heart failure (27), revealing a protective role of FXI in cardiac damage that diverges from its conventional role in coagulation. These examples demonstrate that the liver-heart axis can modulate immune function and cardiovascular outcomes in various ways, and our study advances the understanding of its regulatory role in transplant immunology.

EVs are a key mechanism by which the liver (and other organs) can exert distal effects. They serve as interorgan communication vectors that transfer complex information in the form of proteins and nucleic acids. Importantly, EVs in transplantation have been shown to have context-dependent effects: EVs can propagate alloimmune activation by carrying donor MHC antigens to recipient antigen-presenting cells, thereby stimulating T cell responses (the so-called “semi-direct” pathway of allorecognition) (28, 29). On the other hand, certain EVs can induce tolerance; for example, EVs derived from tolerogenic DCs or Tregs have been used in experimental models to suppress rejection and promote long-term graft acceptance (30, 31). Thus, EVs represent a double-edged sword in transplant immunity. Their impact (immunogenic versus tolerogenic) hinges on their cellular origin and cargo. In our findings, the liver-derived EVs, which carry a cargo that includes ATIII, appear to fall on the immunoregulatory side, contributing to a less inflammatory environment in the rejecting heart. This suggests a scenario in which, during acute rejection, the host liver senses systemic inflammation or tissue injury signals and responds by secreting protective EVs aimed at dampening the immune response.

To test the functional necessity of the EV pathway, we inhibited EV biogenesis using GW4869. The results revealed a complex biological picture. Immunologically, blocking EV secretion unleashed a systemic and local hyperinflammatory state, confirming that endogenous EVs act as a physiological brake on immune activation. However, this aggravated inflammatory profile did not translate into shortening of graft survival. This dissociation between the inflammatory phenotype and the survival outcome likely reflects the biological complexity of global pharmacological inhibition in a high-stringency rejection model. Two primary factors may account for this survival equivalence. First, the nonspecific nature of GW4869 results in a global suppression of EV-mediated signaling. It is plausible that the loss of protective hepatic EVs, which carry cargoes such as ATIII, was partially offset by the simultaneous inhibition of proinflammatory EVs derived from activated immune cells or the graft itself. The effect on graft survival may thus represent a counterbalance between the loss of protective signals and the suppression of inflammatory propagation. Second, allograft rejection is driven by redundant pathways. When the vesicular channel is impaired, the immune system may compensate through EV-independent pathways, such as direct cell-cell contact or cytokine-driven signaling, to maintain the kinetics of graft destruction.

ATIII is traditionally recognized as a pivotal guardian of hemostatic balance; however, its potent antiinflammatory properties suggest a broader role in modulating transplant outcomes (15, 32–34). Our proteomic analysis revealed a marked upregulation of ATIII within plasma EVs during acute cardiac rejection. We characterized this phenomenon as an adaptive “compensatory response” by the host liver, aimed at counteracting the prothrombotic and proinflammatory milieu inherent to alloimmune attack. The physiological necessity of this response was substantiated by our loss-of-function studies, in which targeted silencing of hepatic *Serpinc1* transformed the rejection process into a fulminant failure. These data identify basal hepatic ATIII output as a critical physiological brake on alloimmunity. Mechanistically, our findings define a highly orchestrated signaling axis in which ATIII functions as an upstream regulator of HO-1. Intriguingly, the spatial pattern of *Hmox1* expression provides critical insights into the topology of this interorgan crosstalk. Our in vivo tracing confirmed the physical homing of hepatocyte-derived EVs to the heart, whereas ATIII-driven HO-1 induction was strictly confined to the liver, with cardiac *Hmox1* levels remaining stable across all interventions. This spatial dissociation suggests a dual-mode protective mechanism. Systemically, the ATIII-dependent induction of hepatic HO-1 appears to foster a cytoprotective and antiinflammatory milieu that dampens the systemic alloimmune response. Locally, hepatocyte-derived EVs, potentially shuttling ATIII and other cargo, may deliver these protective signals directly to the graft microenvironment, where they contribute to the suppression of localized proinflammatory cascades and chemokine networks (e.g., *Il1b, Tnfa, Cxcl9/10*). However, the precise molecular mechanisms underlying this local EV action remain to be fully defined, representing an important focus for future investigation.

We acknowledge that although our AAV strategy extended survival, it did not achieve the indefinite tolerance reported with high-dose recombinant ATIII (32, 35). This discrepancy in efficacy is likely attributable to divergent pharmacokinetic profiles. Recombinant protein therapy typically achieves immediate, high-potency systemic effects through supraphysiological bolus dosing, albeit at the risk of hemorrhage. In contrast, our AAV-mediated approach establishes a continuous, liver-driven source of ATIII that mimics natural secretion kinetics. Furthermore, the incremental benefit from gene transfer is inherently limited by the preexisting endogenous ATIII response. As evidenced by our knockdown models, this natural defense is functional and essential. Consequently, recombinant protein offers a potent acute intervention, whereas AAV-mediated delivery provides a more homeostatic approach to transplant immunomodulation, representing a distinct and clinically relevant strategy for long-term graft protection.

In light of our findings, we also evaluated the potential of exogenous EV adoptive transfer. However, injection of exogenous EVs failed to confer a survival benefit comparable to our AAV-mediated approach (Supplemental Figure 7 and Supplemental Table 8), which may be attributed to several interconnected limitations. Rapid clearance by the mononuclear phagocyte system likely shortens the systemic half-life of injected EVs, reducing their opportunity to reach the cardiac allograft in sufficient quantities. Moreover, our mCherry trafficking experiments revealed that even endogenous EVs accumulate only minimally in the heart, suggesting that exogenously delivered EVs also exhibit insufficient targeting efficiency. Consequently, the injection regimen employed here, even with a loading maintenance dose design, appears inadequate to overcome these pharmacokinetic and biodistribution barriers and establish a sustained therapeutic effect within the allograft. Together, these data underscore the unique advantages of a sustained endogenous hepatic EV pathway. Our AAV8 model essentially transforms the host liver into a biological factory, enabling stable and long-term EV secretion that more faithfully recapitulates interorgan communication under physiological conditions, thereby overcoming the pharmacokinetic and targeted-delivery challenges associated with exogenous EV administration. Future studies may further enhance the efficacy of exogenous EV therapy through EV engineering (e.g., improving cardiac tropism) or by combining with sustained-release delivery systems.

The last but not least aspect worth discussing is why the liver’s EV response is vigorously triggered during rejection. Acute rejection of a heart graft leads to tissue injury that likely releases danger signals (damage-associated molecular patterns) into the circulation. The liver, which filters blood, may sense these signals (such as heme from hemolyzed cells, cytokines from activated leukocytes, or circulating donor MHC peptides) and respond by altering its secretory profile. The increase in ATIII levels within circulating EVs could be one manifestation of an acute-phase response aimed at immune modulation.

Despite the clear trends we observed, this study has several limitations. First, our model is a heterotopic (non-working) cardiac transplant in rodents, which is well-established for studying rejection but does not fully recapitulate the hemodynamic stresses of orthotopic transplantation in a large animal or human. The immunological insights are valuable, but translating dosing and efficacy of ATIII augmentation to a clinical scenario will require further studies, potentially in orthotopic transplant models or nonhuman primates. Regarding experimental design, this study exclusively utilized male animals to ensure a stable baseline for proteomic analysis and mechanistic evaluation. Although this strategy minimized confounding factors during the discovery phase, we acknowledge that the efficacy of the ATIII-mediated liver-heart axis remains to be characterized in female cohorts to ensure full clinical relevance. Lastly, our proteomic approach could not capture miRNAs or other nonprotein cargo in EVs, which might also play roles. Although our AAV models validate the hepatic ATIII axis, plasma EV protection extends beyond EV-ATIII alone. Proteomics revealed other rejection-enriched liver proteins (Hgfac, Serping1, Saa4) that likely synergize with ATIII. This multi-cargo requirement explains why adoptively transferred ATIII-EVs failed to prolong survival, further hampered by rapid clearance and poor cardiac tropism. Therefore, maintaining a functional circulating pool to suppress remote inflammation requires a continuous liver-driven supply, justifying our AAV-mediated approach.

In conclusion, our study demonstrates that the liver plays an active immunoregulatory role in acute cardiac allograft rejection. Hepatocyte-derived EVs are key mediators of this interorgan crosstalk, with ATIII acting as a critical effector that drives the protective response via an anticoagulant-independent mechanism involving HO-1 induction. Therapeutically, bolstering this hepatic pathway via AAV-mediated overexpression suppresses intragraft inflammation and extends survival. These findings collectively define the liver as a remote regulator of transplant immunity and suggest that targeting hepatic-to-graft signaling offers a potent strategy for extending allograft survival.

## Methods

### Sex as a biological variable.

Our current study examined male rats only. Our study utilized male rats exclusively to ensure biological homogeneity during the initial discovery phase. Sex hormones, specifically estrogen, are recognized as potent modifiers of T cell responses and allograft survival (36–38). Utilizing a single sex cohort was necessary to maximize the signal-to-noise ratio in the proteomic dataset and identify primary signatures before expanding to female models in future translational validation.

### Animal experimental design and rat model of heart transplantation.

The study employed 3 experimental groups: the sham, Iso, and Allo groups. In the Allo group, full MHC-mismatched heart transplants were performed by transplanting Brown Norway donor hearts into Lewis recipients to induce acute rejection. In contrast, the Iso group received Lewis donor hearts transplanted into MHC-identical Lewis recipients. The sham group underwent a sham surgical procedure on Lewis rats, which mimicked the operative process but without cardiac transplantation. Recipients in all groups were euthanized 5 days after transplantation for subsequent analysis. A schematic of the study design is shown in Figure 1A.

Male Lewis (strain code 113, 4–5 weeks old) and Brown Norway (strain code 113, 4–5 weeks old) rats were obtained as donors, and male Lewis rats (strain code 113, 9–10 weeks old) served as recipients. All animals were purchased from Vital River Laboratory Animal Technology. Rats were housed under specific pathogen–free conditions with constant temperature (22°C) and humidity and fed commercial rat chow pellets. Cervical heterotopic heart transplantation was performed using a technique adapted from previous study (39). Briefly, donor cardiac allografts were transplanted into the recipient’s neck by anastomosing the donor aorta and pulmonary artery to the recipient’s external carotid artery and external jugular vein, respectively. Cardiac graft function was assessed daily via neck palpation, with rejection defined as the day of palpable heartbeat cessation. Grafts failing within 48 hours after transplantation were classified as technical failures, and corresponding animals were excluded from the study. On postoperative day 5, recipient plasma was collected for EV isolation and subsequent analysis. Donor heart tissue was also harvested at this time point for histological analysis.

### Histology and IHC analysis.

The harvested cardiac grafts were fixed in 10% formalin, followed by paraffin embedding and sectioning at 5 μm thickness. Then, sections were subjected to H&E staining. Acute allograft rejection was graded in accordance with the International Society for Heart and Lung Transplantation classification scheme (40).

For IHC analysis, serial sections were immunostained using the following rabbit monoclonal primary antibodies from Abcam: anti-CD4 (clone CAL4; ab237722), anti-CD8 (clone CAL66; ab237709), and anti-CD68 (clone EPR23917-164; ab283654).

### EV purification from plasma samples.

EVs were isolated from plasma samples using ultracentrifugation. Briefly, thawed plasma samples were centrifuged at 2,000*g* for 30 minutes at 4°C to remove cellular debris. The supernatant was carefully transferred to new tubes and subjected to 10,000*g* centrifugation for 45 minutes at 4°C to eliminate larger vesicles. After passing the resulting supernatant through a 0.45 μm membrane filter to remove residual particles, the filtrate underwent ultracentrifugation at 100,000*g* for 70 minutes at 4°C (SW41 rotor, Beckman Coulter). The pellet was then resuspended in 10 mL of ice-cold 1× PBS and purified by repeating ultracentrifugation under identical parameters. Finally, the EV-enriched pellet was resuspended in 100 μL of cold 1× PBS and stored at −80°C for downstream analyses.

### Electron microscopy of EVs.

EV samples were adsorbed onto copper grids and negatively stained with 2% phosphotungstic acid for 10 minutes. After this step, the specimen was dried at room temperature for 2 minutes, and transmission electron microscopy analysis was performed using a Hitachi instrument (JEM-2100 Plus, JEOL) for structural characterization.

### Nanoparticle tracking analysis.

Exosomes were diluted in filtered PBS to a concentration of 10^7^–10^9^ particles/mL. Nanoparticle tracking analysis was performed using the ZetaView system (Particle Metrix) to determine the size distribution and concentration.

### Western blot analysis.

Tissue samples and EV pellets were lysed in ice-cold lysis buffer containing protease inhibitors (Abclonal; RM02916) and 1 mM PMSF (Beyotime; ST507). After centrifugation at 12,000*g* for 20 minutes at 4°C, supernatants were collected for protein quantification using a BCA assay kit (Beyotime; P0012). Protein extracts were denatured in SDS-PAGE loading buffer at 100°C for 10 minutes, resolved on SDS-PAGE gels, and transferred to PVDF membranes (MilliporeSigma; IPFL00010).

Membranes were blocked with 5% nonfat milk in TBS for 2 hours and incubated overnight at 4°C with primary antibodies (1:1,000) against TSG101 (28283-1-AP), CD63 (25682-1-AP), and CD9 (20597-1-AP) from Proteintech; CD81 (R381296) from Zenbio; and ATIII (A11249), β-actin (AC038), and GAPDH (A19056) from Abclonal. After washing with TBST (TBS plus 0.1% Tween 20), membranes were incubated with HRP-conjugated goat anti-rabbit IgG (H+L) secondary antibody (Abclonal; AS014) for 1 hour at 37°C. Proteins were visualized using an ECL kit (TIANGEN; PA112).

### Protein preparation and digestion.

Protein extraction was performed using SDT lysis buffer (4% SDS, 100 mM Tris-HCl, pH 7.6), quantified via BCA assay. Aliquots of 20 μg protein per sample were denatured in 5× loading buffer at 95°C for 5 minutes, then resolved on 4%–20% gradient SDS-PAGE gels (180 V, 45 min). After Coomassie blue R-250 staining for quality control, filter-aided sample preparation was implemented. Proteins were reduced with 10 mM DTT (37°C, 1.5 h), alkylated with 20 mM iodoacetamide in the dark (30 min), and transferred to 10 kDa cutoff ultrafiltration units. After sequential washing with 8 M urea and 25 mM NH_4_HCO_3_ buffer, tryptic digestion proceeded overnight at 37°C. Digested peptides were desalted on C18 cartridges (Empore, 3 mL bed volume), vacuum-dried, and reconstituted in 0.1% formic acid for LC-MS/MS analysis. Peptide concentration was determined by UV absorbance at 280 nm, applying an extinction coefficient of 1.1 for 0.1% (g/L) protein solutions.

### LC-MS/MS analysis.

Peptide samples were analyzed using a timsTOF Pro mass spectrometer (Bruker Daltonics) coupled to a NanoElute nanoflow HPLC system. Chromatographic separation was achieved on a C18 reversed-phase analytical column (25 cm × 75 μm ID, 1.9 μm particles; Thermo Fisher Scientific) at 300 nL/min with a 60-minute linear gradient from 95% buffer A (0.1% formic acid in water) to 60% buffer B (0.1% formic acid in acetonitrile). During separation, the mass spectrometer operated in positive ion mode with a 1.5 kV electrospray voltage.

Data acquisition employed parallel accumulation-serial fragmentation (PASEF) mode across *m/z* 100–1,700 and ion mobility coefficients (1/K_0_) of 0.6–1.6 Vs/cm². Each acquisition cycle comprised 1 full MS scan followed by 10 PASEF MS/MS scans, implementing a 24-second dynamic exclusion window. Raw data were processed using MaxQuant (v1.6.14; Max Planck Institute of Biochemistry) for protein identification and label-free quantification against the UniProt Rattus norvegicus (Rat) proteome database.

### Bioinformatic analysis.

Identified proteins were cross-referenced with the extracellular vesicle proteome database Vesiclepedia (v5.1, 2023, http://www.microvesicles.org/) (17) to validate EV associations. DEPs were identified using the limma R package under the criteria of *P* less than 0.05 and |log_2_(fold change)| greater than 1. GO and KEGG pathway analyses were subsequently performed with the clusterProfiler package (v4.5.0) in R (v4.2.2) (41) through Hiplot Pro (https://hiplot.com.cn/), a comprehensive web service for biomedical data analysis and visualization.

Protein organ-specificity was classified based on Human Protein Atlas (https://www.proteinatlas.org/) transcriptomic criteria, in which tissue-enriched proteins exhibit at least 4-fold higher mRNA levels in single tissues versus others, group-enriched proteins show at least 4-fold elevation in 2–5 tissue clusters, and tissue-enhanced proteins demonstrate at least a 4-fold increase relative to overall averages. Protein origins validated through this approach are detailed in Table 1, with results visualized via ggplot2.

Protein-protein interaction networks of candidate DEPs were constructed using the STRING database (https://string-db.org/), followed by topological analysis in Cytoscape (v3.10.0) with the CytoHubba plugin applying the maximal clique centrality algorithm for hub gene identification.

### Palm-mCherry labeling of EVs.

To produce mCherry-labeled EVs, a palmitoylation (palm) motif was inserted in-frame with the N-terminus of mCherry following an established protocol (19, 21). This lipid modification mediates the anchoring of proteins to cellular membranes via covalent thioester bonds between cysteine residues and palmitate. The recombinant palm-mCherry fusion protein was thus directed to the plasma membrane. To achieve hepatocyte-specific expression, AAV8 vectors carrying the palm-mCherry construct under the control of the thyroid hormone-binding globulin (TBG) promoter were delivered to 5-week-old male C57BL/6 mice (Vital River Laboratory Animal Technology, strain code 219) via tail vein injection at a dose of 2 × 10¹¹ vector genomes (vg). The AAV8-TBG-palm-mCherry was produced and purified commercially by BrainVTA Co., Ltd.

### In vivo modulation of hepatic ATIII expression and EV inhibition.

To investigate the role of ATIII (*Serpinc1*) in liver-mediated immune regulation, liver-specific genetic manipulation was performed using recombinant AAV8 vectors under the control of the TBG promoter.

For ATIII overexpression, AAV8 vectors encoding rat *Serpinc1* cDNA (AAV-Serpinc1) and control vectors encoding GFP (AAV-GFP) were obtained from Angel Biotech. Lewis recipient rats received a single tail vein injection of 3.5 × 10^12^ vg suspended in 200 μL of PBS.

For ATIII knockdown, AAV8 vectors expressing a shRNA targeting rat *Serpinc1* (AAV-shSerpinc1) and nontargeting scrambled control vectors (AAV-shCtrl) were synthesized by Obio Technology. The target sequence for *Serpinc1* was 5′-CGUUAGUGAGAUUGUCUAU-3′. Rats in the knockdown cohort were administered 1 × 10^12^ vg via the caudal vein.

In both genetic models, vector administration was performed 4 weeks prior to heart transplantation to ensure stable transgene expression or silencing. The efficiency of ATIII overexpression or knockdown in liver tissues was verified by Western blot analysis at the experimental endpoint.

To suppress the secretion of EVs in vivo, the neutral sphingomyelinase-2 (nSMase2) inhibitor GW4869 (Selleck Chemicals; S7609) was administered following previously established protocols (19, 21). Briefly, recipient rats received i.p. injections of GW4869 (1.25 mg/kg). The initial dose was administered 1 hour before heart transplantation, followed by daily injections until the experimental endpoint.

### Plasma hematological and biochemical parameter analysis.

Peripheral blood samples collected in EDTA-coated tubes were subjected to complete blood count analysis using a hematology analyzer. For biochemical profiling, lithium heparin-anticoagulated plasma samples were separated via centrifugation and assayed on an SMT-120V automated biochemical analyzer (Seamaty) following the manufacturer’s instructions.

### RT-qPCR.

Total RNA was extracted using TRIzol reagent (Invitrogen; 15596018), followed by cDNA synthesis with a PrimeScript RT reagent kit with gDNA Eraser (Takara Bio; RR047A). qPCR amplification was performed on a LineGene 9600 Real-Time PCR System (Bioer Technology) using TB Green Premix Ex Taq (Takara Bio; RR420A). Relative mRNA expression levels were quantified via the 2^(–ΔΔCt)^ method, normalized to *Gapdh*. Primers are listed in Supplemental Table 9.

### Statistics.

Data are expressed as mean ± SEM and were analyzed via GraphPad Prism (v10.4.1). Two-group comparisons used 2-tailed, unpaired Student’s *t* tests or Mann-Whitney *U* test; multiple groups were compared by 1-way ANOVA with Tukey’s post hoc test. Graft survival was assessed by Kaplan-Meier curves and log-rank tests. *P* less than 0.05 was considered significant.

### Study approval.

All animal experiments were approved by the IACUC of Soochow University (approval 202504A0787). All animal husbandry and experiments were strictly carried out in accordance with the Guide for the Care and Use of Laboratory Animals (National Academies Press, 2011).

### Data availability.

Values for all data points in graphs are reported in the Supporting Data Values file. All raw data that support the findings of this study are available from the corresponding author upon reasonable request. The mass spectrometry proteomics data have been deposited to the ProteomeXchange Consortium (https://proteomecentral.proteomexchange.org) via the iProX partner repository (42, 43) with the dataset identifier PXD072924.

## Author contributions

SD, FW, and ZS conceived the study. SD and FW designed the experiments. SD, W. Zhou, FC, and HZ performed the majority of the experiments and acquired the data. XZ, JH, and SJ participated in experiments. ZJ and FC supervised the technical aspect of the transplantation model. SD and W. Zhang performed bioinformatic analyses. SD, W. Zhou, and HZ analyzed data and edited figures. SD and FW wrote the original draft. ZS reviewed and edited the manuscript. SD, FW, and ZS provided funding. FW and ZS provided supervision. All authors critically reviewed the manuscript and accepted the final version of the manuscript. The order of co–first and co–senior authors was decided on the basis of the time and effort of their relative contributions made toward the project and manuscript. All authors approved of this order.

## Conflict of interest

The authors have declared that no conflict of interest exists.

## Funding support

- National Natural Science Foundation of China (grants 92168203 and 82241201 to ZS; grant 82400513 to FW; grant 82500468 to SD).
- Special Fund for Basic Research of Jiangsu Province (Soft Science Research) Project (grant BK20240795 to FW).
- Jiangsu Cardiovascular Medicine Innovation Center (grant CXZX202210 to the Institute for Cardiovascular Science, Soochow University).
- Natural Science Foundation of Jiangsu Province (grant BK20240363 to SD).
- Suzhou Basic Research Pilot Project (grant SSD2024064 to SD).

## Supplementary Material

Supplemental data

Unedited blot and gel images

Supporting data values

## Figures and Tables

**Table 1:**
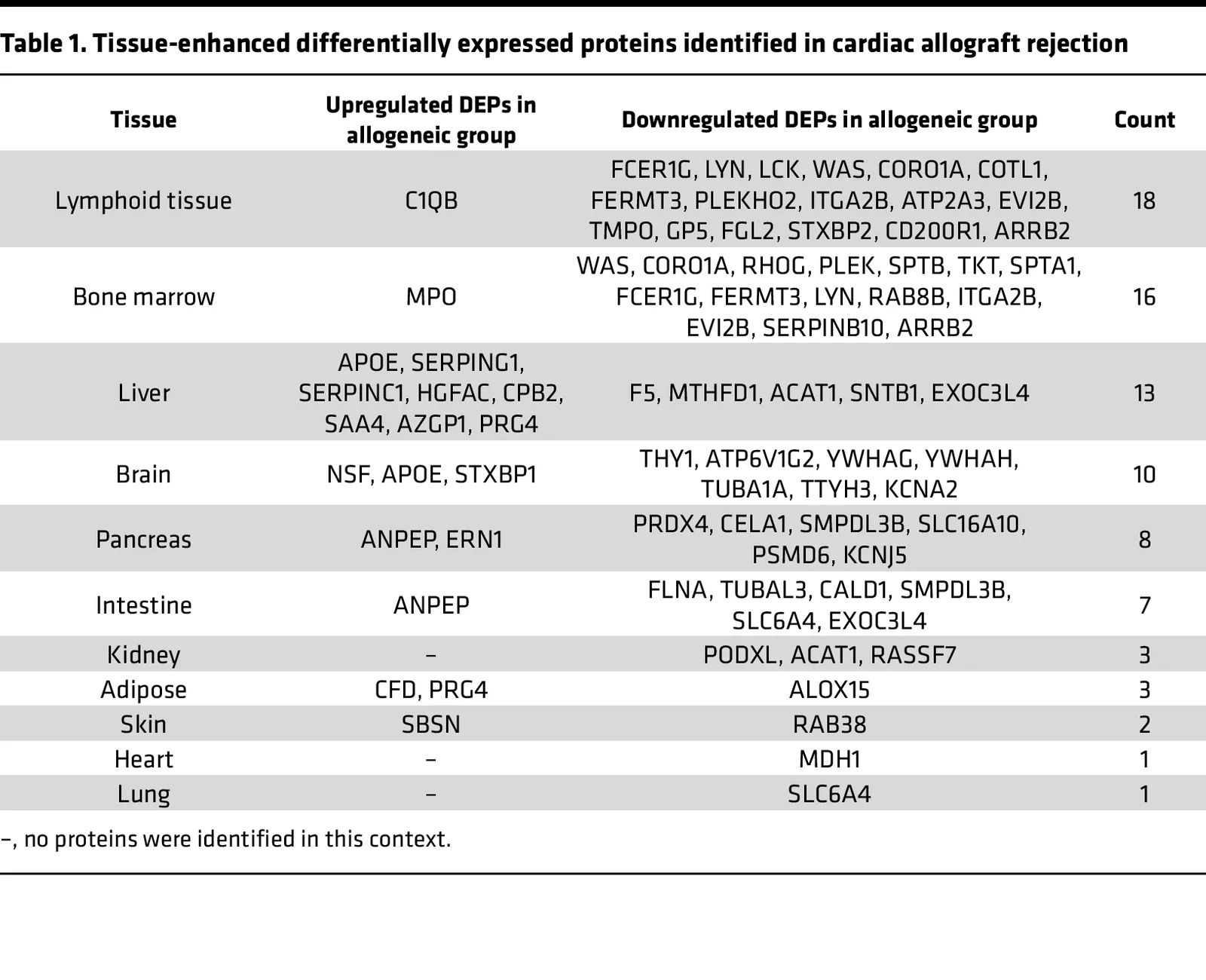
Tissue-enhanced differentially expressed proteins identified in cardiac allograft rejection

## Version 1 07/02/2026

In-Press Preview

## Version 2 08/24/2026

Electronic publication
