# Supplementary material for: Proteomic profiling of plasma extracellular vesicles reveals a therapeutically targetable liver-heart axis in cardiac transplantation
Source: JCI Insight. 2026 Jul 2;11(16):e200422. doi: 10.1172/jci.insight.200422 (PMC13502184; doi:10.1172/jci.insight.200422)
Supplement: Supplementary file 1 [file jciinsight-11-200422-s015.pdf]

Supplementary information

Proteomic Profiling of Plasma Extracellular Vesicles Reveals a Therapeutically Targetable Liver-Heart Axis in Cardiac Transplantation

Shiyu Dai[1#*]; Wei Zhou[1#]; Fangyu Chen[1#]; Huanyu Zhang[1#]; Zhenchun Ji[1]; Xuejing Zong[1]; Wanruo Zhang[1]; Jie Hu[1]; Shumin Jiang[1]; Fei Wang[1*]; Zhenya Shen[1*]

[1]Institute for Cardiovascular Science & Department of Cardiovascular Surgery of the First Affiliated Hospital, Soochow University, Suzhou, Jiangsu 215123, China.

[#]Shiyu Dai, Wei Zhou, Fangyu Chen, and Huanyu Zhang contributed equally to this work.

Major Correspondence:

Zhenya Shen, M.D., Ph.D.

Soochow University, Suzhou, China

Email: uuzyshen@aliyun.com

Co-Correspondence:

Fei Wang, Ph.D.

Soochow University, Suzhou, China

Email: wf@suda.edu.cn

Co-Correspondence:

Shiyu Dai, Ph.D.

Soochow University, Suzhou, China

Email: 15629039156@163.com

This file includes:

Supplementary methods;

Supplementary figures 7;

Supplementary tables 9.

Supplementary methods

Proteinase K protection assay

To determine the sub-vesicular localization of ATIII, a protease protection assay was performed on purified plasma EVs. Briefly, equal amounts of isolated EVs were divided into three aliquots and subjected to different enzymatic treatments: (i) an untreated control incubated with PBS vehicle to establish baseline protein levels; (ii) Proteinase K treatment, in which EVs were incubated with Proteinase K (200 μg/mL) to degrade proteins exposed on the external surface; and (iii) Proteinase K plus detergent treatment, where EVs were incubated with Proteinase K in the presence of 1% Triton X-100 to disrupt the vesicular membranes and expose luminal contents to proteolysis. All samples were incubated at 37℃ for 30 min, and the reaction was subsequently terminated by the addition of 5 mM phenylmethylsulfonyl fluoride (PMSF) followed by incubation on ice for 10 min. After denaturation in loading buffer at 95℃ for 5 min, the protein profiles were analyzed via Western blotting. To ensure assay validity, CD9 and TSG101 were simultaneously detected as representative markers for surface-exposed and luminal-protected proteins, respectively.

Adoptive transfer of plasma-derived extracellular vesicles (EVs)

To evaluate the immunomodulatory effects of ATIII-enriched EVs, donor Lewis rats were intravenously injected with AAV-Serpinc1 or AAV-GFP (control) at a dose of 3.5×10^{12} vector genomes (vg) per rat. Four weeks post-injection, plasma-derived EVs were isolated from these donor rats via differential ultracentrifugation.

For the adoptive transfer experiments, recipient male Lewis rats (8–10 weeks old) received an initial loading dose of 200 μg of purified EVs (resuspended in 200 μL PBS) via tail vein injection 2 hours prior to the initiation of cardiac transplantation. Following the surgery, maintenance doses of

100 μg were administered every other day via the tail vein until the experimental endpoint. The dosage and administration frequency were determined based on previously reported dosing regimens in EV-based therapeutics (1-3). Post-transplantation, recipient rats were randomly assigned to two study cohorts. In the survival cohort, allograft function was monitored daily via manual palpation, with graft failure defined as the complete cessation of palpable heartbeats. In the pathological cohort, rats were euthanized on post-operative day 5 (POD 5) to harvest cardiac grafts for subsequent histological and pathological evaluation.

Statistics

Data are presented as mean ± standard error of the mean (SEM) and analyzed using GraphPad Prism 10.4.1 Software. For comparisons between two experimental groups, a two-tailed unpaired Student's *t*-test was employed. For comparisons among three or more groups, one-way analysis of variance (ANOVA) was used, followed by Tukey's multiple comparisons test. The significance threshold was established at $P < 0.05$.

References

1. Barile L, et al. Extracellular vesicles from human cardiac progenitor cells inhibit cardiomyocyte apoptosis and improve cardiac function after myocardial infarction. *Cardiovasc Res*. 2014;103(4):530-541.
2. Gupta D, et al. Dosing extracellular vesicles. *Adv Drug Deliv Rev*. 2021;178:113961.
3. Wang J, et al. Extracellular vesicles mediate the communication of adipose tissue with brain and promote cognitive impairment associated with insulin resistance. *Cell Metab*. 2022;34(9):1264-1279 e8.

Supplementary figures

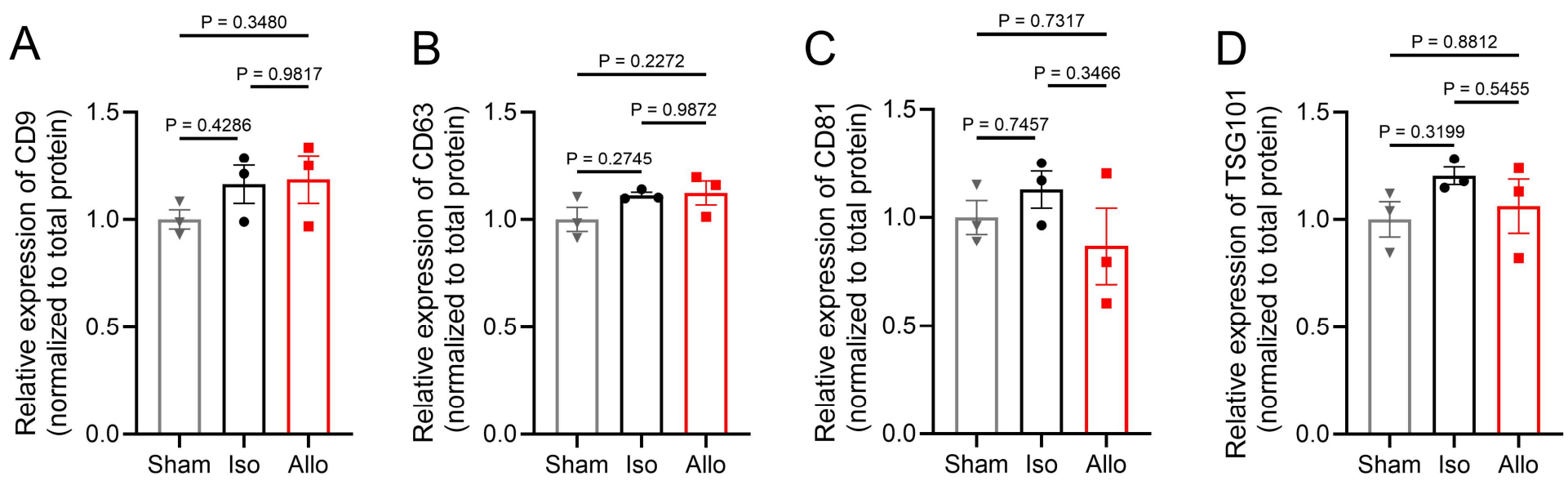

Supplementary Figure 1. Quantification of EV marker protein expression.

Densitometric analysis of (A) CD9, (B) CD63, (C) CD81, and (D) TSG101 protein bands from Western blots shown in Figure 1F. Band intensities were quantified and normalized to the total protein signal from the corresponding lane as determined by Ponceau S staining of the same membrane. Data are presented as mean ± SEM (n = 3 biologically independent samples per group: allogeneic group [Allo], isogeneic group [Iso], and sham-operated controls [Sham]). Statistical significance was determined by one-way ANOVA followed by Tukey's multiple comparisons test.

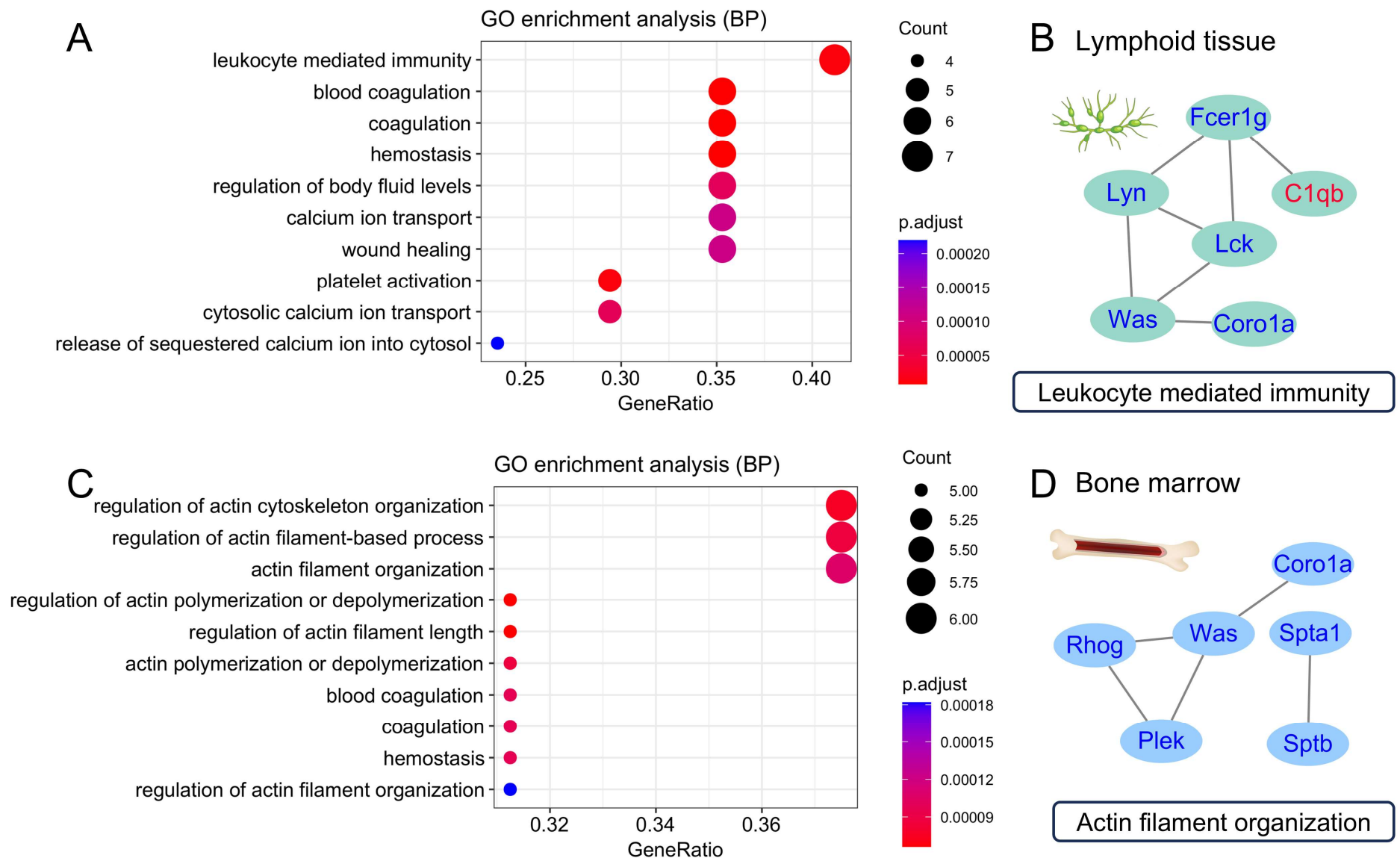

Supplementary Figure 2. Characterization of circulating EV proteins derived from lymphoid and bone marrow tissues during cardiac rejection.

(A) Functional enrichment in lymphoid tissue. Gene Ontology biological process (GO-BP) analysis of differentially expressed proteins (DEPs) enhanced in lymphoid tissues. (B) Integrated visualization of lymphoid tissue-enhanced proteins and associated signaling cascades (leukocyte-mediated immunity) modulated in allograft recipients. (C) Functional enrichment in bone marrow. GO-BP analysis of DEPs enhanced in bone marrow tissues. (D) Representation of bone marrow-enhanced proteins and relevant signaling cascades (actin filament organization) altered during rejection. In panels (B) and (D), label colors indicate protein expression changes in the Allo group compared with the Sham group: blue denotes down-regulated proteins, red denotes up-regulated proteins. Enrichment *P* values were calculated by hypergeometric test and BH-adjusted (FDR < 0.05).

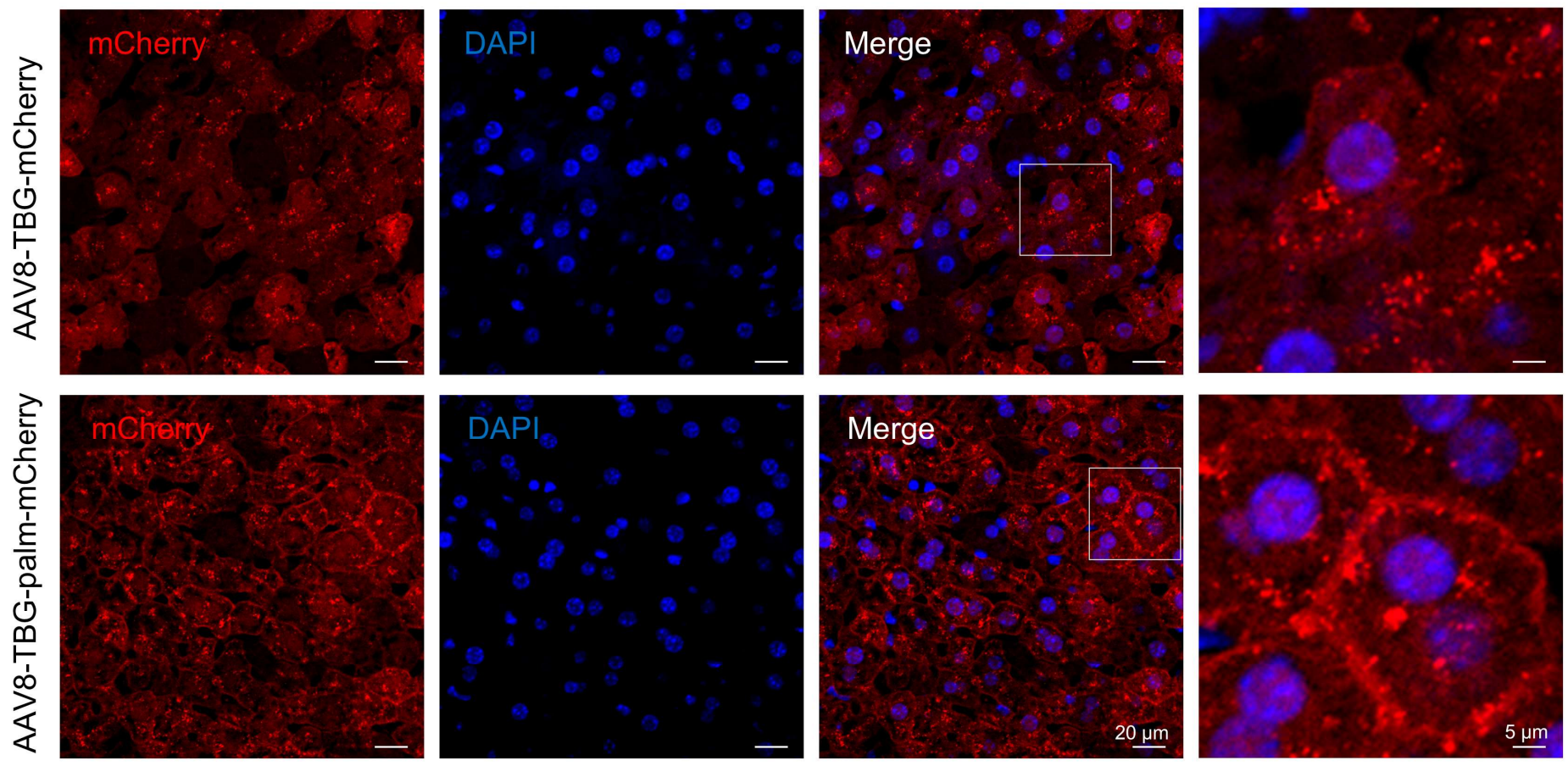

Supplementary Figure 3. Validation of hepatocyte-specific AAV transduction.
Representative fluorescence images of liver tissues. Four weeks following tail vein injection of AAV8-TBG-palm-mCherry or AAV8-TBG-mCherry, robust and widespread mCherry fluorescence (red) was observed across the hepatic parenchyma. The high-magnification view (magnified from the boxed area) demonstrates the characteristic membrane-localized expression of mCherry within hepatocytes, consistent with the design of the palm-mCherry reporter for EV labeling. Images are representative of n = 3 mice per group. Scale bars: 20 μm (left panel) and 5 μm (right magnified view).

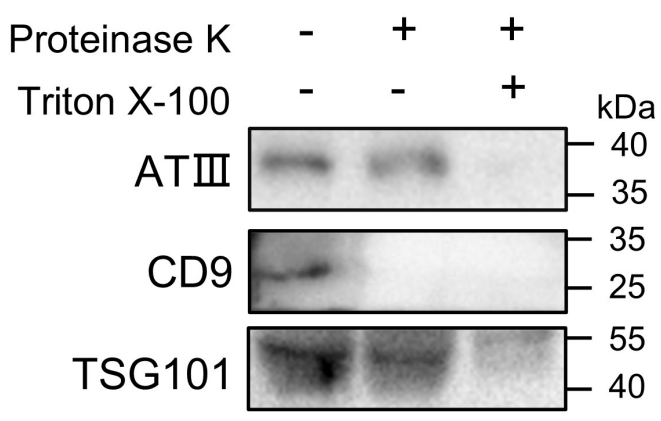

Supplementary Figure 4. ATIII is localized within the EV lumen.
Western blot analysis of ATIII in purified EVs treated with Proteinase K (PK) with or without 1% Triton X-100. CD9 and TSG101 served as surface and luminal controls, respectively. Blots are representative of two independent experiments.

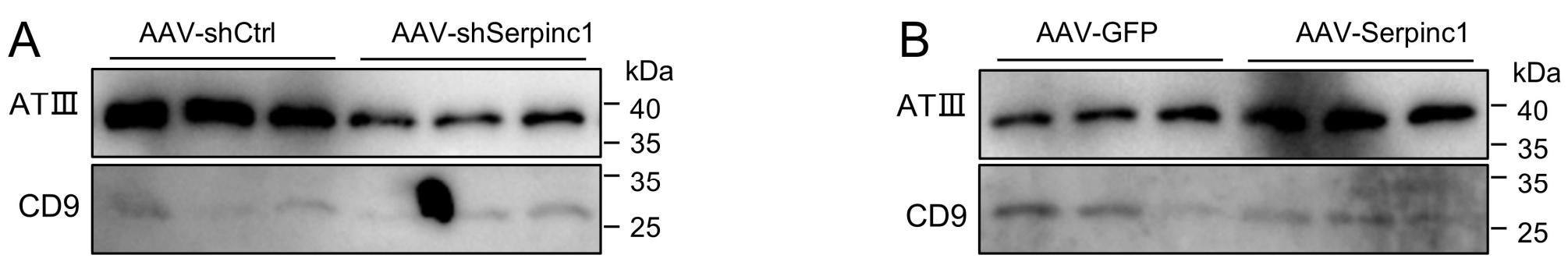

Supplementary Figure 5. Verification of ATIII protein levels in plasma-derived EVs.
Representative Western blots showing ATIII abundance in plasma EVs isolated from recipients following (A) AAV-mediated hepatic ATIII knockdown (AAV-shSerpinc1 vs. shCtrl) and (B) AAV-mediated hepatic ATIII overexpression (AAV-Serpinc1 vs. AAV-GFP). CD9 served as a loading control. Blots shown are from three biological replicates (n = 3) per group.

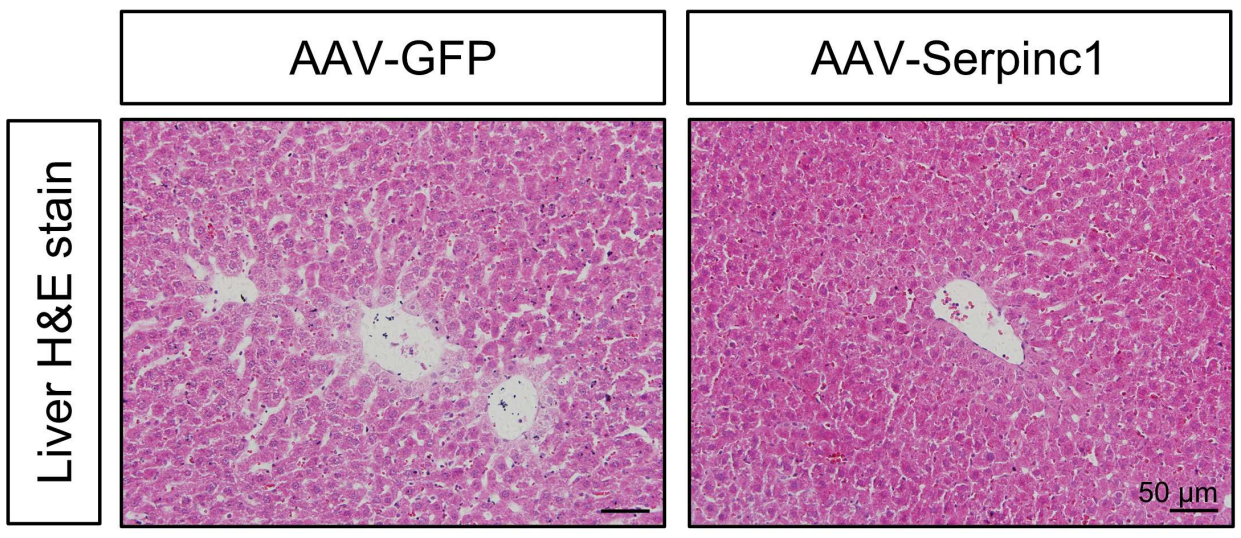

Supplementary Figure 6. Histological assessment of recipient liver safety.

Representative H&E staining of liver sections from recipients treated with AAV-GFP (n = 4) and AAV-Serpinc1 (n = 5). Scale bar: 50 μm.

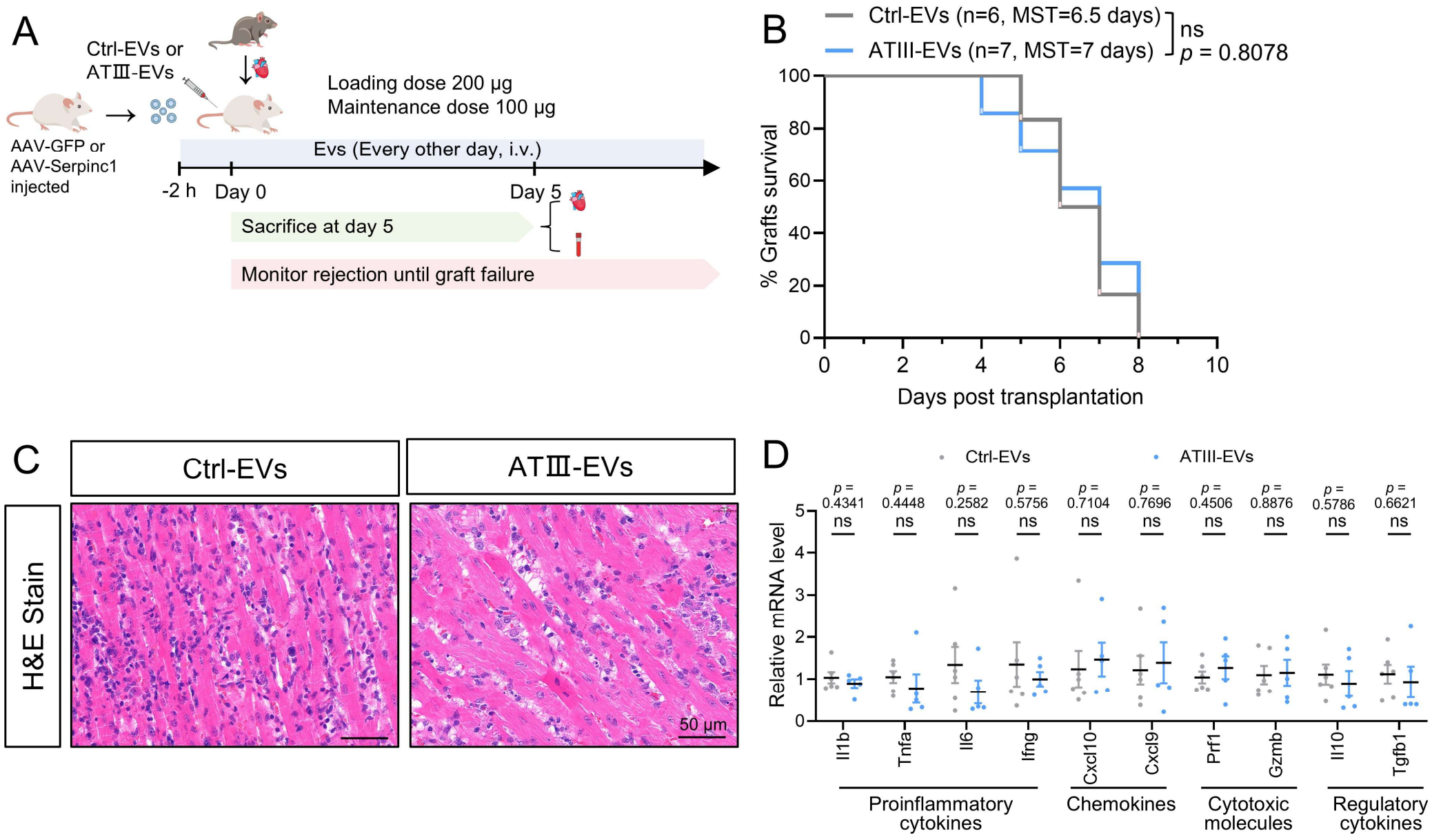

Supplementary Figure 7. Impact of exogenous ATIII-enriched EV adoptive transfer on cardiac allograft rejection.

(A) Experimental workflow. Recipient Lewis rats were pre-conditioned with ATIII-EVs or Control-EVs (200 μg, i.v.) 2 hours prior to transplantation, followed by maintenance doses (100 μg, i.v., every other day). (B) Kaplan-Meier curves comparing allograft survival between groups. Log-rank test (Ctrl-EVs, n = 6; ATIII-EVs, n = 7). (C) Representative H&E staining of cardiac allografts from the biological replicates. Scale bar: 50 μm. (D) Graft inflammatory profile. Relative mRNA levels of proinflammatory cytokines (*Il1b, Tnfa, Il6, Ifng*), chemokines (*Cxcl9, Cxcl10*), cytotoxic molecules (*Prf1, Gzmb*), and regulatory factors (*Tgfb1, Il10*) in cardiac allografts. Data are presented as mean ± SEM. Sample sizes for (C and D): Ctrl-EVs, n = 6; ATIII-EVs, n = 5. Unpaired Student's *t*-test determined statistical significance for comparisons in (D). ns, not significant.

Supplementary Table 1. Quantitative profiling and comparative analysis of liver-derived EV proteins in plasma.

Gene	LFQ intensity			Allo/Sham		Allo/Iso		Iso/sham	
Name	Sham	Iso	Allo	Fold change	*P* Value	Fold change	*P* Value	Fold change	*P* Value
Hgfac	94844.33	192126.67	348490.00	3.67	0.02*	1.81	0.04*	2.03	0.13
Serping1	2058633.33	3105833.33	6462600.00	3.14	0.03*	2.08	0.07	1.51	0.23
Saa4	1159543.33	2281666.67	3371900.00	2.91	0.01*	1.48	0.01*	1.97	0.07
Serpinc1	5467300.00	7122866.67	15652333.33	2.86	0.02*	2.20	0.03*	1.30	0.27
Azgp1	533583.33	387370.00	1490466.67	2.79	0.04*	3.85	0.02*	0.73	0.49
Cpb2	583763.33	971573.33	1486300.00	2.55	0.04*	1.53	0.24	1.66	0.29
Prg4	47789.33	88018.67	103456.67	2.16	0.04*	1.18	0.69	1.84	0.32
Apoe	16231733.33	23090000.00	33615333.33	2.07	0.02*	1.46	0.13	1.42	0.33
F5	7695333.33	5368266.67	2852833.33	0.37	0.02*	0.53	0.32	0.70	0.41
Mthfd1	48279.00	41551.00	16233.00	0.34	0.02*	0.39	0.07	0.86	0.44
Acat1	132774.00	35847.00	19624.33	0.15	0.04*	0.55	0.03*	0.27	0.06
Sntb1	53673.07	4343.77	--	--	--	--	--	0.08	0.14
Exoc3l4	45104.67	17125.00	--	--	--	--	--	0.38	0.39

--, No peptides were identified in this context. *$P < 0.05$.

Supplementary Table 2. Plasma hematological analysis of recipients in Vehicle- and GW4869-treated groups.

Variables	Vehicle	GW4869	*P* value
WBC, 10^9/L	2.44 ± 1.09	4.26 ± 0.96	0.010**
LYM#, 10^9/L	1.09 ± 0.41	2.19 ± 0.6	0.003**
MON#, 10^9/L	0.36 ± 0.22	0.45 ± 0.24	0.539
NEU#, 10^9/L	0.98 ± 0.51	1.62 ± 0.58	0.063
LYM%, %	46.23 ± 7.32	52.00 ± 12.28	0.342
MON%, %	13.86 ± 4.06	10.24 ± 4.15	0.153
NEU%, %	39.19 ± 6.15	37.5 ± 9.62	0.724
HGB, g/L	146.71 ± 26.95	150.14 ± 28.22	0.833
RBC, 10^{12}/L	7.48 ± 1.21	7.67 ± 1.22	0.792
HCT, %	38.26 ± 6.05	39.3 ± 6.41	0.777
MCV, fL	51.2 ± 1	51.23 ± 0.54	0.952
MCH, pg	19.54 ± 0.5	19.49 ± 0.71	0.874
MCHC, g/L	382.29 ± 13.67	380.57 ± 13.25	0.829
RDW-SD, fL	25.6 ± 1.81	25.8 ± 1.22	0.786
RDW-CV, %	12.47 ± 0.7	12.57 ± 0.41	0.766
PLT, 10^9/L	378.43 ± 56.61	369 ± 41.76	0.876
PCT, %	0.29 ± 0.03	0.28 ± 0.03	0.981
MPV, fL	7.67 ± 0.71	7.74 ± 0.45	1.000
PDW, %	13.50 ± 4	15.28 ± 2.99	0.580

Values represent means ± SD (Vehicle, n = 7; GW4869, n = 7).

WBC, White blood cell count; LYM#, Lymphocyte count; MON#, Monocyte count; NEU#, Neutrophils count; LYM%, Lymphocyte percentage; MON%, Monocyte percentage; NEU%, Neutrophils percentage; HGB, Hemoglobin; RBC, Red blood cell count; HCT, Hematocrit; MCV, Mean corpuscular volume; MCH, Mean corpuscular hemoglobin; MCHC, Mean corpuscular hemoglobin concentration; RDW-SD; Red cell distribution width-standard Deviation; RDW-CV, Red cell distribution width-coefficient of variation; PLT, Platelet count; PCT, Plateletcrit; MPV, Mean platelet volume; PDW, Platelet distribution width. ***P* < 0.01 vs. Vehicle-treated group.

Supplementary Table 3. Plasma biochemical parameter analysis of recipients in Vehicle- and GW4869-treated groups.

**Variables**	**Vehicle**	**GW4869**	***P* value**
Albumin, g/L	28.68 ± 2.43	26.80 ± 1.02	0.141
Total protein, g/L	57.40 ± 6.32	53.53 ± 2.12	0.224
Globulin, g/L	28.72 ± 4.34	26.73 ± 1.22	0.348
Albumin/Globulin ratio	1.01 ± 0.11	1.01 ± 0.03	0.878
Total bilirubin, μmol/L	0.98 ± 0.58	2.05 ± 0.50	0.051
AST, U/L	205.00 ± 167.43	170.33 ± 176.73	0.757
ALT, U/L	61.33 ± 13.49	50.83 ± 5.87	0.142
Amylase, U/L	1201.17 ± 103.55	1007.5 ± 135.24	0.029*
Creatine kinase, U/L	351.17 ± 175.95	283.33 ± 226.31	0.608
Creatinine, μmol/L	32.93 ± 6.84	38.53 ± 9.12	0.298
Urea nitrogen, mmol/L	6.24 ± 1.23	5.65 ± 0.78	0.383
Urea nitrogen/Creatinine ratio	201.22 ± 69.34	153.04 ± 33.14	0.191
Glucose, mmol/L	12.09 ± 1.21	11.72 ± 1.36	0.661
Triglycerides, mmol/L	1.18 ± 0.47	0.88 ± 0.47	0.341
Calcium, mmol/L	2.50 ± 0.13	2.44 ± 0.09	0.424
Inorganic phosphorus, mmol/L	2.20 ± 0.14	2.30 ± 0.24	0.426
Calcium and phosphorus product, $mmol^2/L^2$	5.49 ± 0.50	5.60 ± 0.53	0.731

Values represent means ± SD (Vehicle, n = 6; GW4869, n = 6).

AST, aspartate aminotransferase; ALT, alanine aminotransferase. *$P < 0.05$ vs. Vehicle-treated group.

Supplementary Table 4. Plasma hematological analysis of recipients in AAV-shCtrl and AAV-shSerpinc1 groups.

Variables	AAV-shCtrl	AAV-shSerpinc1	*P* value
WBC, 10^9/L	2.60 ± 1.11	4.14 ± 1.12	0.054
LYM#, 10^9/L	1.12 ± 0.40	2.15 ± 0.63	0.012*
MON#, 10^9/L	0.56 ± 0.18	0.57 ± 0.15	0.951
NEU#, 10^9/L	0.92 ± 0.59	1.42 ± 0.62	0.215
LYM%, %	44.32 ± 7.67	51.85 ± 6.62	0.127
MON%, %	22.40 ± 3.01	15.02 ± 5.47	0.025*
NEU%, %	32.87 ± 7.57	32.88 ± 6.39	0.997
HGB, g/L	132.17 ± 8.01	140.50 ± 18.89	0.385
RBC, 10^{12}/L	7.14 ± 0.38	7.36 ± 0.82	0.602
HCT, %	35.05 ± 1.76	36.98 ± 4.45	0.388
MCV, fL	49.10 ± 0.57	50.18 ± 0.65	0.019*
MCH, pg	18.52 ± 0.25	19.08 ± 0.47	0.038*
MCHC, g/L	377.00 ± 4.97	380.00 ± 6.61	0.436
RDW-SD, fL	24.35 ± 0.26	24.90 ± 0.16	0.001**
RDW-CV, %	12.30 ± 0.15	12.28 ± 0.11	0.845
PLT, 10^9/L	408.17 ± 58.95	429.25 ± 36.69	0.783
PCT, %	0.30 ± 0.04	0.31 ± 0.03	0.665
MPV, fL	7.42 ± 0.33	7.25 ± 0.09	0.894
PDW, %	13.07 ± 2.92	10.08 ± 0.71	0.366

Values represent means ± SD (AAV-shCtrl, n = 6; AAV-shSerpinc1, n = 6).
WBC, White blood cell count; LYM#, Lymphocyte count; MON#, Monocyte count; NEU#, Neutrophils count; LYM%, Lymphocyte percentage; MON%, Monocyte percentage; NEU%, Neutrophils percentage; HGB, Hemoglobin; RBC, Red blood cell count; HCT, Hematocrit; MCV, Mean corpuscular volume; MCH, Mean corpuscular hemoglobin; MCHC, Mean corpuscular hemoglobin concentration; RDW-SD; Red cell distribution width-standard Deviation; RDW-CV, Red cell distribution width-coefficient of variation; PLT, Platelet count; PCT, Plateletcrit; MPV, Mean platelet volume; PDW, Platelet distribution width. *$P < 0.05$, **$P < 0.01$ vs. AAV-shCtrl group.

Supplementary Table 5. Plasma biochemical parameter analysis of recipients in AAV-shCtrl and AAV-shSerpinc1 groups.

Variables	AAV-shCtrl	AAV-shSerpinc1	*P* value
Albumin, g/L	26.70 ± 0.64	27.15 ± 2.80	0.758
Total protein, g/L	46.90 ± 16.60	54.80 ± 4.89	0.331
Globulin, g/L	27.60 ± 1.24	27.65 ± 2.23	0.969
Albumin/Globulin ratio	0.97 ± 0.05	0.98 ± 0.04	0.704
Total bilirubin, µmol/L	2.05 ± 1.11	1.53 ± 1.07	0.622
AST, U/L	217.83 ± 154.42	230.83 ± 202.55	0.911
ALT, U/L	34.00 ± 13.08	45.83 ± 9.25	0.129
Amylase, U/L	898.33 ± 360.45	1122.33 ± 160.59	0.233
Creatine kinase, U/L	533.17 ± 622.63	203.17 ± 60.12	0.265
Creatinine, µmol/L	32.40 ± 11.56	38.75 ± 7.77	0.332
Urea nitrogen, mmol/L	4.45 ± 1.78	5.31 ± 0.27	0.312
Urea nitrogen/Creatinine ratio	137.59 ± 61.01	142.17 ± 27.72	0.882
Glucose, mmol/L	9.78 ± 3.82	11.60 ± 1.97	0.366
Triglycerides, mmol/L	1.51 ± 0.57	1.11 ± 0.34	0.222
Calcium, mmol/L	2.13 ± 0.59	2.46 ± 0.07	0.248
Inorganic phosphorus, mmol/L	1.85 ± 0.74	2.04 ± 0.35	0.616
Calcium and phosphorus product, $mmol^2/L^2$	4.37 ± 1.89	5.01 ± 0.91	0.510

Values represent means ± SD (AAV-shCtrl, n = 6; AAV-shSerpinc1, n = 6).
AST, aspartate aminotransferase; ALT, alanine aminotransferase.

Supplementary Table 6. Plasma hematological analysis of recipients in AAV-GFP and AAV-Serpinc1 groups.

Variables	AAV-GFP	AAV-Serpinc1	*P* value
WBC, 10^9/L	3.25 ± 0.62	5.09 ± 2.46	0.230
LYM#, 10^9/L	0.72 ± 0.51	1.14 ± 0.95	0.474
MON#, 10^9/L	0.30 ± 0.12	0.73 ± 0.38	0.051
GRA#, 10^9/L	2.24 ± 0.60	3.21 ± 1.74	0.235
LYM%, %	22.40 ± 13.79	20.59 ± 8.24	0.823
MON%, %	8.65 ± 2.66	17.63 ± 12.06	0.376
GRA%, %	68.95 ± 13.17	61.79 ± 13.19	0.861
HGB, g/L	136.15 ± 3.59	154.81 ± 23.99	0.447
RBC, 10^{12}/L	7.20 ± 0.27	8.18 ± 1.29	0.463
HCT, %	38.45 ± 0.99	44.26 ± 6.87	0.377
MCV, fL	53.53 ± 0.74	54.17 ± 0.98	0.199
MCH, pg	18.60 ± 0.46	18.89 ± 0.29	0.949
MCHC, g/L	356.00 ± 6.87	349.43 ± 5.47	0.243
RDW-SD, fL	26.75 ± 0.43	27.14 ± 1.12	0.539
RDW-CV, %	12.45 ± 0.05	12.49 ± 0.44	0.966
PLT, 10^9/L	480.75 ± 77.28	458.80 ± 117.85	0.785
PCT, %	0.47 ± 0.07	0.45 ± 0.11	0.788
MPV, fL	9.85 ± 0.29	9.92 ± 0.32	0.774
PDW, fL	12.38 ± 1.21	12.26 ± 1.14	0.901
PLCR, %	19.05 ± 2.21	19.30 ± 2.45	0.893

Values represent means ± SD (AAV-GFP, n = 4; AAV-Serpinc1, n = 5).

WBC, White blood cell count; LYM#, Lymphocyte count; MON#, Monocyte count; GRA#, Granulocyte count; LYM%, Lymphocyte percentage; MON%, Monocyte percentage; GRA%, Granulocyte percentage; HGB, Hemoglobin; RBC, Red blood cell count; HCT, Hematocrit; MCV, Mean corpuscular volume; MCH, Mean corpuscular hemoglobin; MCHC, Mean corpuscular hemoglobin concentration; RDW-SD; Red cell distribution width-standard Deviation; RDW-CV, Red cell distribution width-coefficient of variation; PLT, Platelet count; PCT, Plateletcrit; MPV, Mean platelet volume; PDW, Platelet distribution width; PLCR, Platelet-large cell ratio.

Supplementary Table 7. Plasma biochemical parameter analysis of recipients in AAV-GFP and AAV-Serpinc1 groups.

**Variables**	**AAV-GFP**	**AAV-Serpinc1**	***P* value**
Albumin, g/L	31.40 ± 0.41	33.28 ± 1.52	0.072
Total protein, g/L	59.18 ± 2.20	62.74 ± 3.33	0.148
Globulin, g/L	27.73 ± 1.88	29.46 ± 1.95	0.273
Albumin/Globulin ratio	1.14 ± 0.07	1.13 ± 0.04	0.899
Total bilirubin, µmol/L	2.00 ± 1.05	1.48 ± 0.25	0.432
AST, U/L	53.75 ± 4.82	60.00 ± 7.13	0.227
ALT, U/L	45.00 ± 4.12	53.20 ± 6.31	0.088
Amylase, U/L	783.00 ± 354.51	1590.80 ± 239.64	0.009**
Creatine kinase, U/L	176.25 ± 67.30	200.20 ± 52.36	0.613
Creatinine, µmol/L	57.25 ± 15.03	39.64 ± 10.16	0.108
Urea nitrogen, mmol/L	4.79 ± 0.57	5.38 ± 1.09	0.408
Urea nitrogen/Creatinine ratio	93.44 ± 39.85	138.27 ± 17.76	0.087
Glucose, mmol/L	12.30 ± 0.82	14.26 ± 2.08	0.161
Triglycerides, mmol/L	1.36 ± 0.22	1.56 ± 0.37	0.427
Calcium, mmol/L	1.26 ± 0.79	2.46 ± 0.60	0.057
Inorganic phosphorus, mmol/L	2.21 ± 0.13	2.64 ± 0.23	0.022*
Calcium and phosphorus product, $mmol^2/L^2$	2.85 ± 1.94	6.51 ± 1.83	0.037*

Values represent means ± SD (AAV-GFP, n = 4; AAV-Serpinc1, n = 5).

AST, aspartate aminotransferase; ALT, alanine aminotransferase. *$P < 0.05$, **$P < 0.01$ vs. AAV-GFP control group.

Supplementary Table 8. Plasma hematological analysis of recipients in Ctrl-EVs and ATIII-EVs-treated groups.

Variables	Ctrl-EVs	ATIII-EVs	*P* value
WBC, 10^9/L	3.09 ± 1.27	3.38 ± 1.70	0.781
LYM#, 10^9/L	1.96 ± 1.16	2.01 ± 1.08	0.948
MON#, 10^9/L	0.33 ± 0.09	0.41 ± 0.21	0.463
NEU#, 10^9/L	0.78 ± 0.19	0.94 ± 0.41	0.471
LYM%, %	60.57 ± 8.69	58.66 ± 1.77	0.673
MON%, %	11.22 ± 2.31	12.12 ± 0.59	0.460
NEU%, %	27.35 ± 6.43	28.26 ± 1.72	0.787
HGB, g/L	123.50 ± 18.12	133.40 ± 13.11	0.380
RBC, 10^{12}/L	6.17 ± 0.97	6.57 ± 0.60	0.480
HCT, %	33.45 ± 4.28	35.80 ± 2.95	0.372
MCV, fL	54.60 ± 2.46	54.52 ± 2.28	0.961
MCH, pg	20.08 ± 0.43	20.30 ± 0.61	0.547
MCHC, g/L	368.17 ± 9.35	372.60 ± 10.09	0.512
RDW-SD, fL	30.18 ± 5.91	27.30 ± 0.22	0.440
RDW-CV, %	13.48 ± 1.90	12.52 ± 0.31	0.336
PLT, 10^9/L	663.50 ± 127.39	578.00 ± 50.80	0.524
PCT, %	0.39 ± 0.06	0.35 ± 0.04	0.545
MPV, fL	5.87 ± 0.23	6.10 ± 0.08	0.567
PDW, fL	6.35 ± 0.36	6.53 ± 0.24	0.492

Values represent means ± SD (Ctrl-EVs, n = 6; ATIII-EVs, n = 5).

WBC, White blood cell count; LYM#, Lymphocyte count; MON#, Monocyte count; NEU#, Neutrophils count; LYM%, Lymphocyte percentage; MON%, Monocyte percentage; NEU%, Neutrophils percentage; HGB, Hemoglobin; RBC, Red blood cell count; HCT, Hematocrit; MCV, Mean corpuscular volume; MCH, Mean corpuscular hemoglobin; MCHC, Mean corpuscular hemoglobin concentration; RDW-SD; Red cell distribution width-standard Deviation; RDW-CV, Red cell distribution width-coefficient of variation; PLT, Platelet count; PCT, Plateletcrit; MPV, Mean platelet volume; PDW, Platelet distribution width.

Supplementary Table 9. Primers used in this study.

Primers	Forward (5’ to 3’)	Reverse (5’ to 3’)
Serpinc1	TCCCAACTCCCAGGGATCAT	GGCCTTGAAGGTCACCCTAC
F2	AATCCAGACCGGGATGAGGA	CGATACCCTTCTCAGCGTCC
F5	TGGAACTCTTCGGCTGTGAC	AATAGCCGCTGTCTCGATGG
F7	GAGCCCTGAGAGAACCAAGC	CACCACTGTGATGTTTACCAATTTC
F8	TTTCAGGGTAACCGGGATGC	AGTGGGGCCATGAATGAGTG
Proc	TCCAGGATGTGGCAGTTCAG	TCTTGTCAGTCCGCTTCCAC
Pros1	TCCTGAGCGTGCGCTTTC	CATTAGCTGACTGCCTCGCA
Thbd	GGGGGTGACTAGATGGGACT	CCCAAGGGAGGTCCCAATTC
SerpinA1	GCTCTGGGACAACAAGTTGAAAA	TAGCCTCTTCTGAGTCGGCA
SerpinG1	GCTGCCTAGTGACCAAGAACT	CCTGTGTGCTCAATGGGACT
F9	AGGCAATTCGCTGGGATTGA	GCAACATGGGGAGCTACACT
F10	CCAATAAAGGACGCGCGAAG	TCATTCAAGATGGTGCCCCC
F12	GGTTTCCCCTGAGAGTCACG	CAAGGACCGACCCTGGTTAC
F11	GCAGGATGACCTCATTACAGCA	GGAGACTGAACCCGGAAAGG
F13b	GTTTTGCCACTGTCGACCAC	ATCCCGTGACCCTTGTAGGA
F13a1	CGCATGTATGTTGCCGTCTG	CGCTGTTTTCTTGGGGTGTG
Serpina10	TTGGCCTGTCTGTGGCTATG	GCCTGTAGGTTGAGCCCATT
Serpind1	CACAACAGCTCAGCACCAAA	TGAATAGGCGATGGGTCAGC
Fgb	GGACAACGATGGCTGGGTAA	CATTCGGATTGGCTGCATGG
Plg	GGCACATCGTCCACTACCAA	AGGGCCCCTCTGGTCATTAT
Fgg	CCAAACAGGTTGGAGACATGTAA	ATCGCCAGCATAAAACTGCT
Fga	CTCTACTGGCACAAGCACCA	AATGCCATTTTGGGCACCTG
Ifng	GGCAAAAGGACGGTAACACG	GTGCTGCATCTGTGGGTTGT
Tnfa	GATTTGGTGACCAGGCTGTC	ATGACCCGTAGGGCGATT
Il6	GGATACCACCCACAACAGAC	TTGCCGAGTAGACCTCATAG
Il10	GACGCTGTCATCGATTTCTCC	TTTCTGGGCCATGGTTCTCT
Il1b	TCATTGTGGCTGTGGAGAAG	CTATGTCCCGACCATTGCTG
Cxcl9	AATCCCTCAAAGACCTCAAACAGT	TCTTCAGTGTAGCGATGATTTCAGT
Cxcl10	CGGTGAGCCAAAGAAGGTCTA	CTAGCCGCACACTGGGTAAA
Tgfb1	AGAGAAGAACTGCTGTGTA	GGTTGTGTTGGTTGTAGAG
Hmox1	TGCACATCCGTGCAGAGAAT	CAGAGTTCACAGCCTCTGGG
Prf1	ATAAGAGTAGTCGTGATTC	CGTAGATGTGATGTAGTC
Gzmb	TCCTTATACGAGAAGACTT	ATGTCATTGGAGATTGTC
Gapdh	AACCTGCCAAGTATGATGA	GGAGTTGCTGTTGAAGTC
