# Supplementary material for: Proteomic profiling of plasma extracellular vesicles reveals a therapeutically targetable liver-heart axis in cardiac transplantation
Source: JCI Insight. 2026 Jul 2;11(16):e200422. doi: 10.1172/jci.insight.200422 (PMC13502184; doi:10.1172/jci.insight.200422)
Supplement: Supplementary file 2 [file jciinsight-11-200422-s016.pdf]

Full unedited gel for Figure 1F

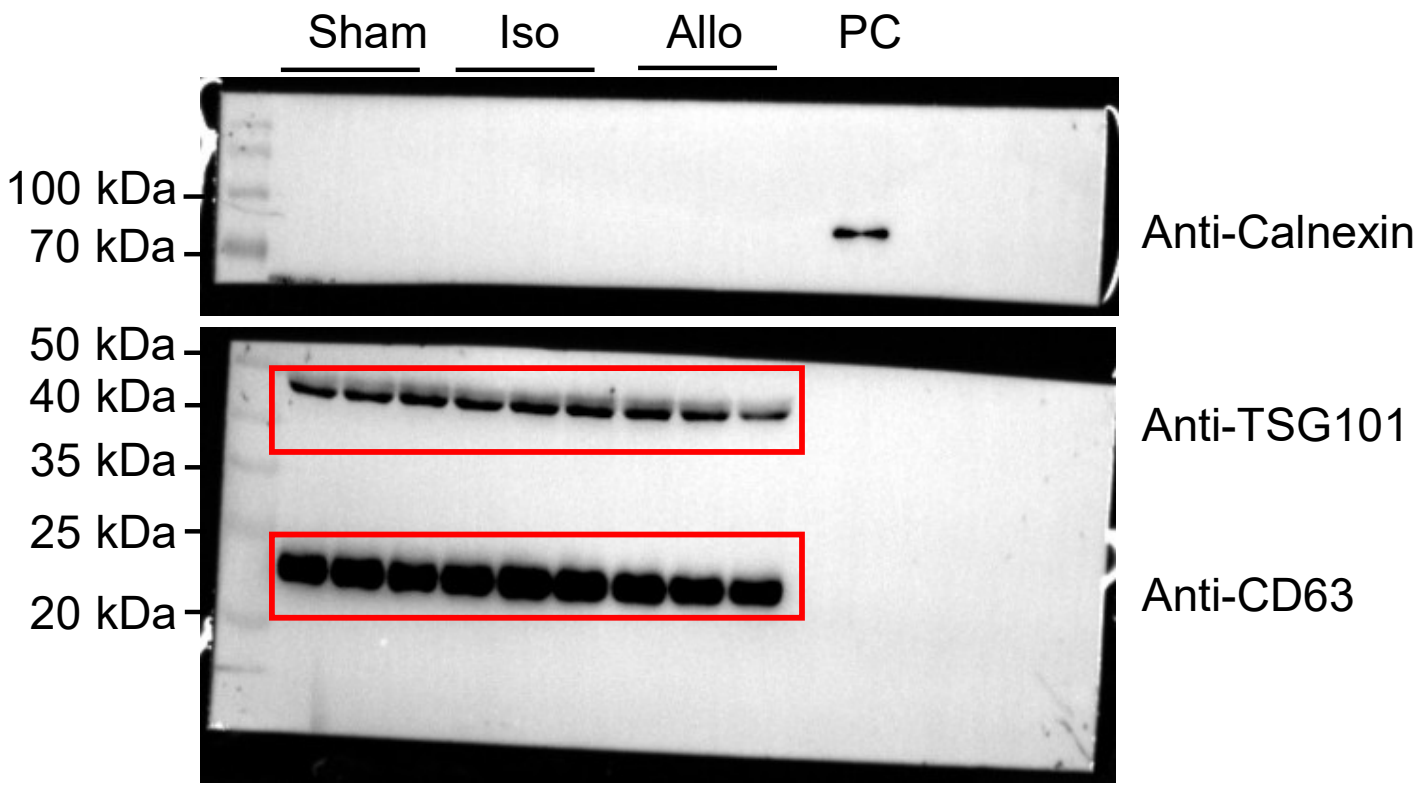

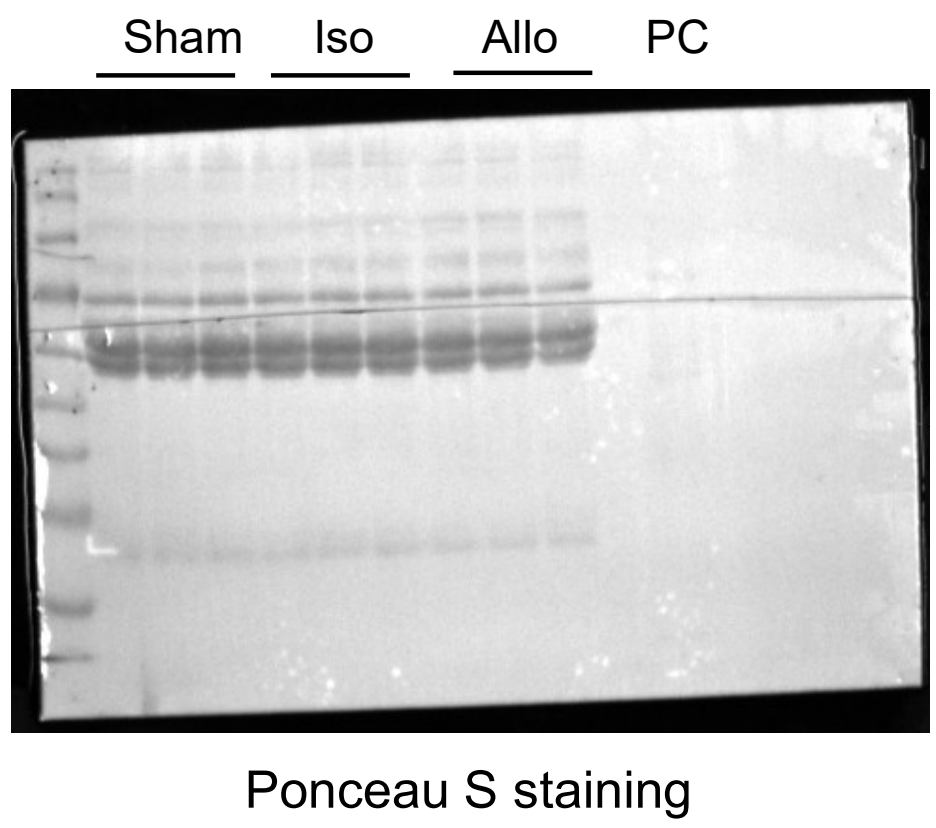

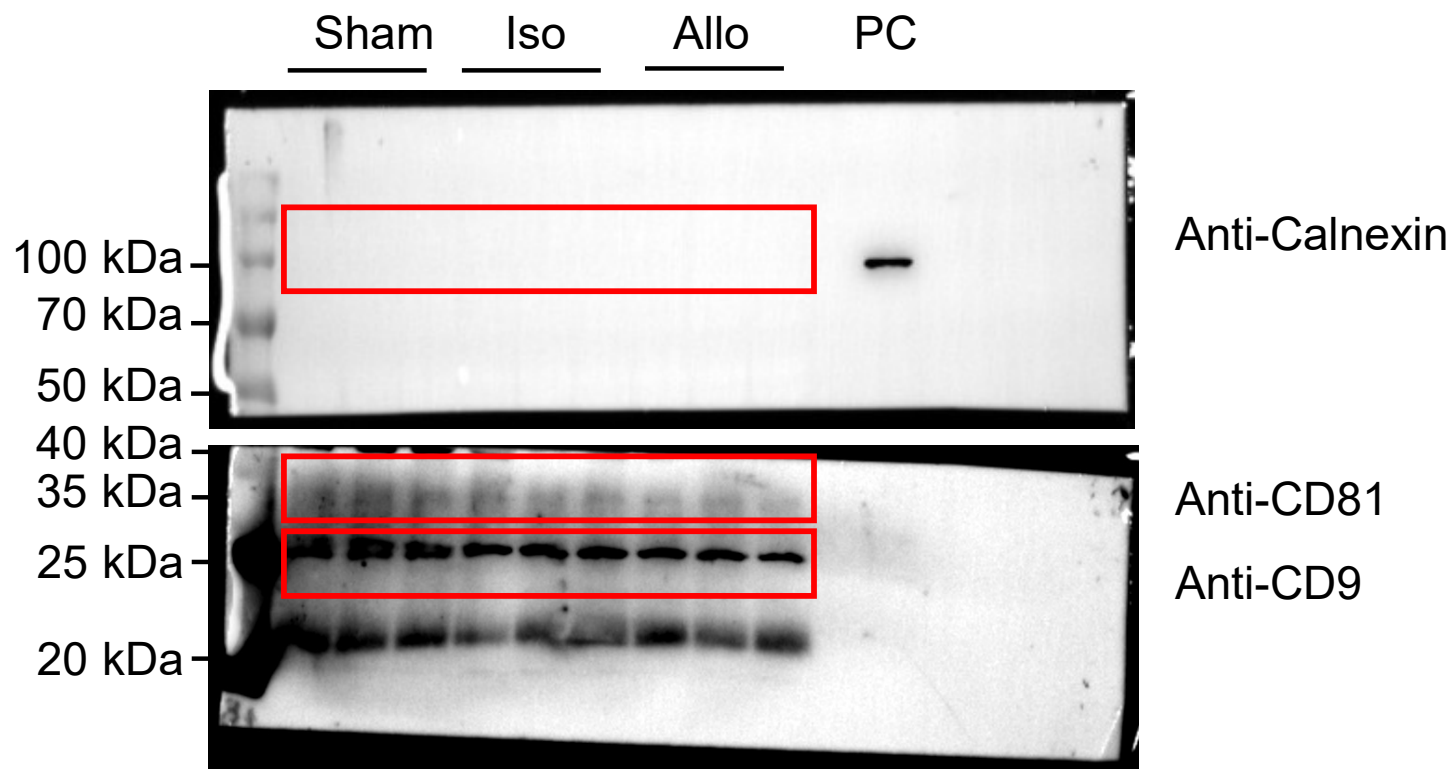

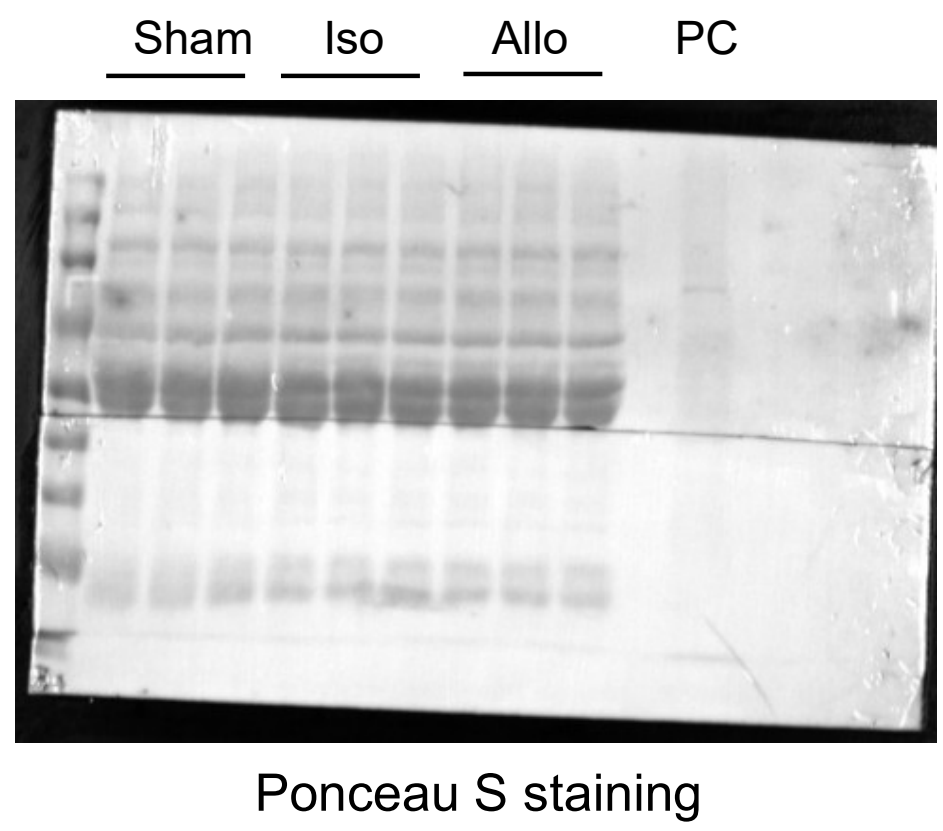

Full unedited gel for Figure 4E

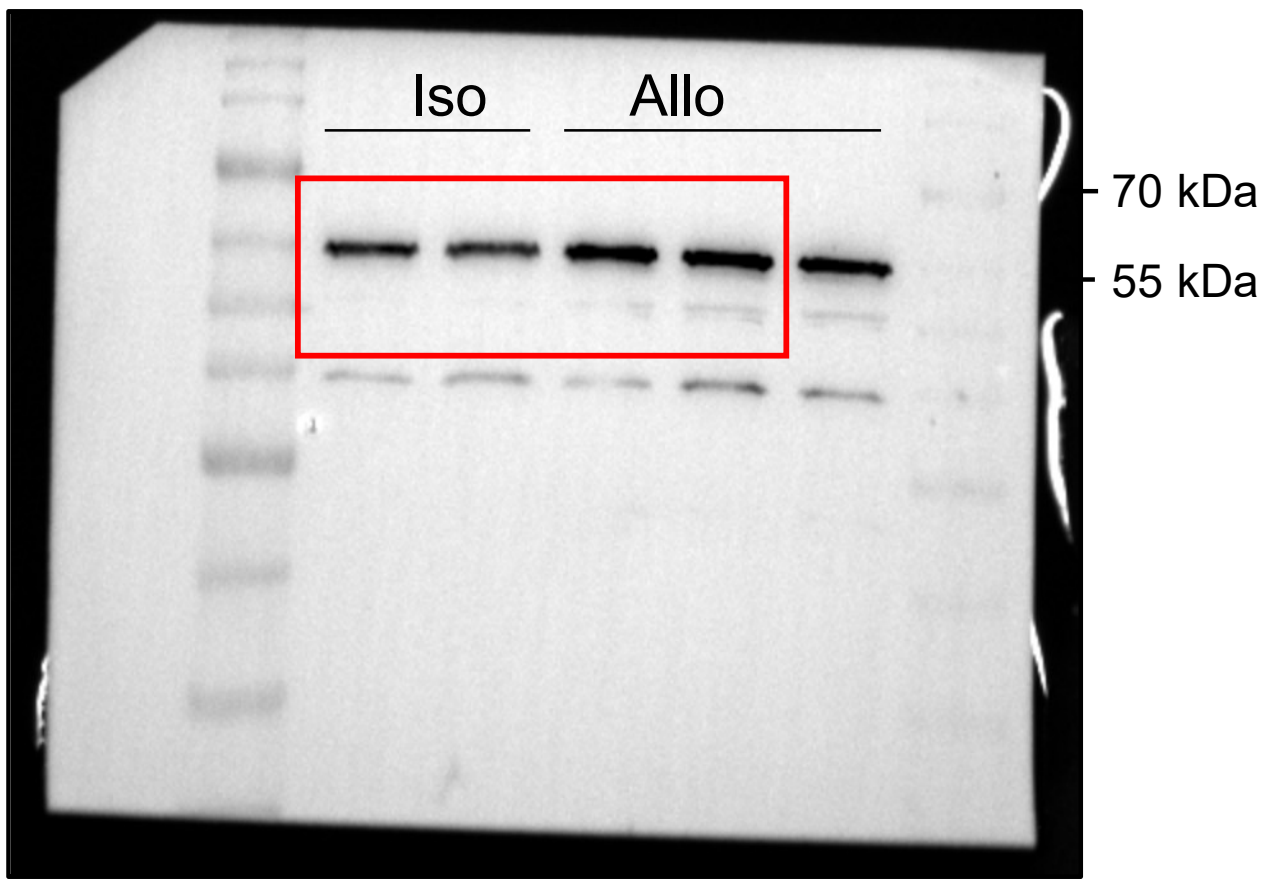

Anti-ATⅢ

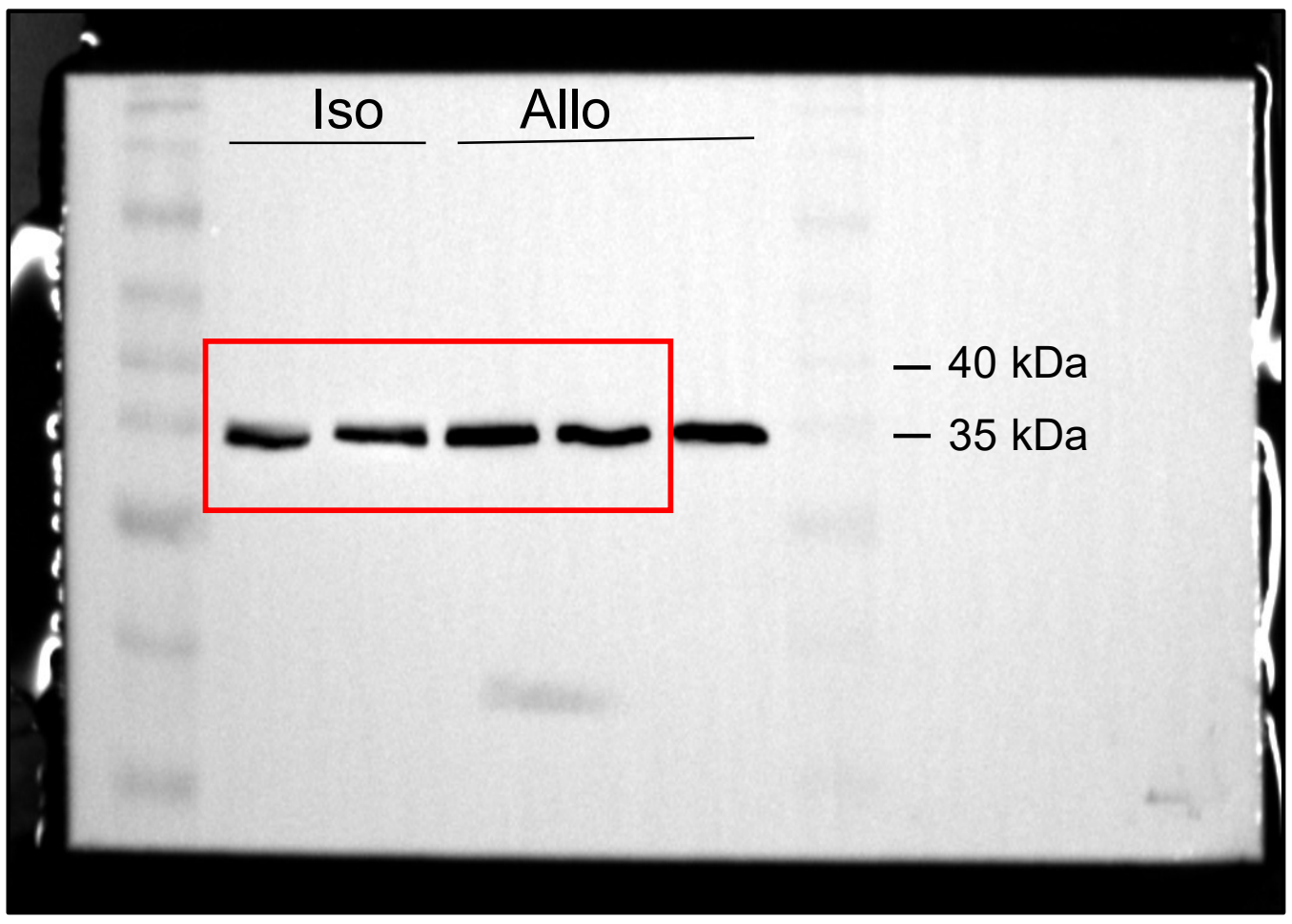

Anti-GAPDH

Full unedited gel for Figure 6C

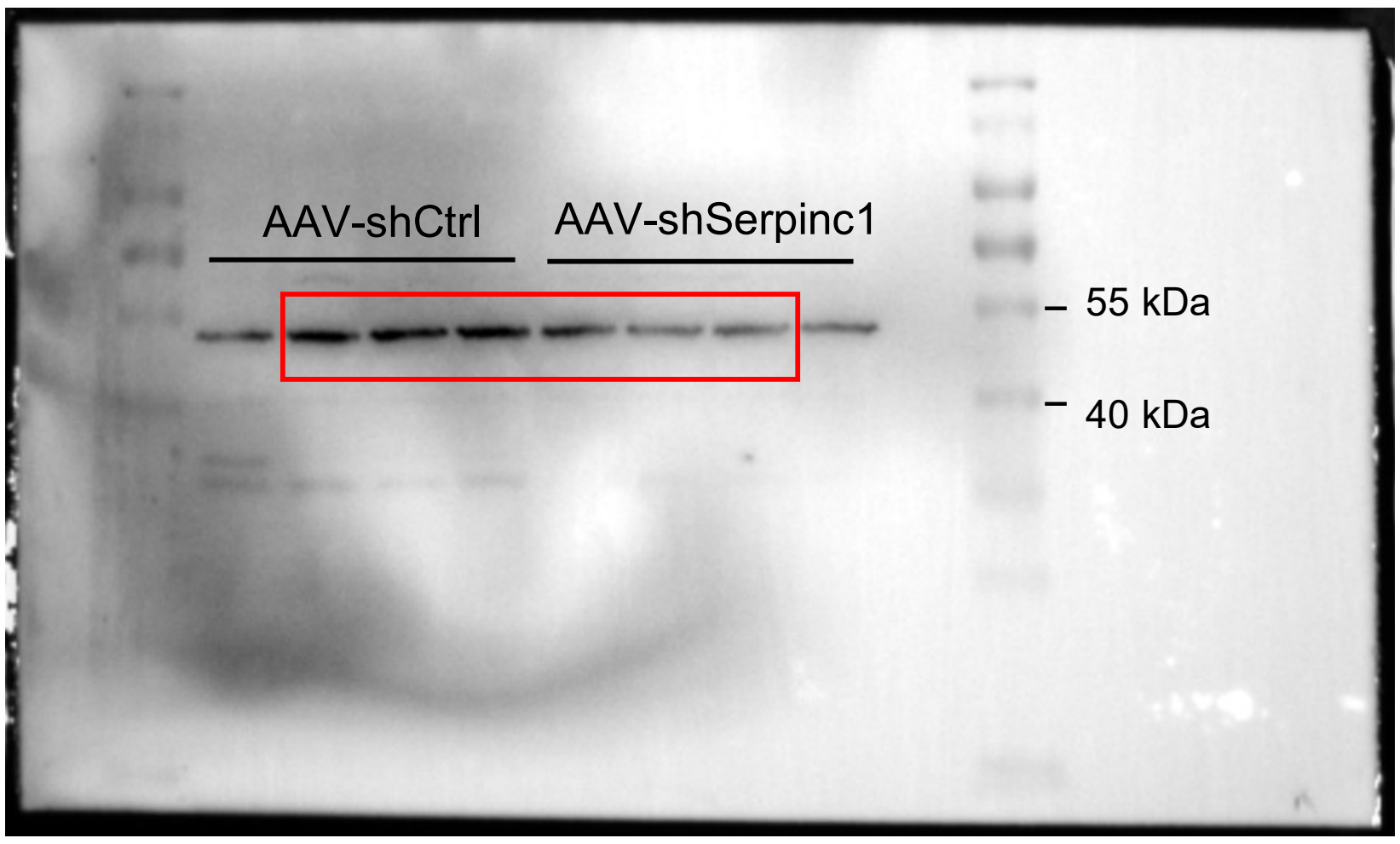

Anti-ATⅢ

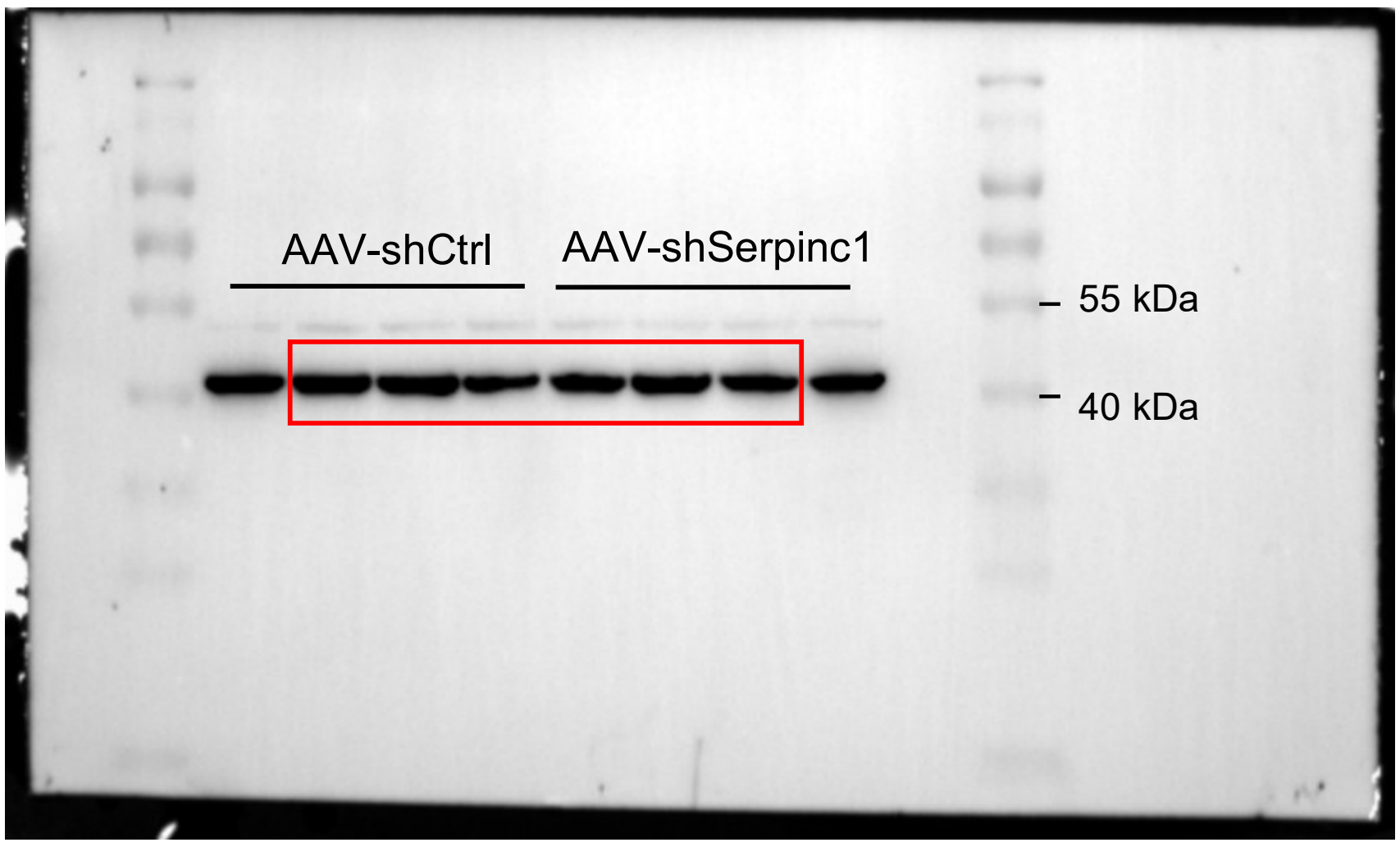

Anti-Actin

Full unedited gel for Figure 7C

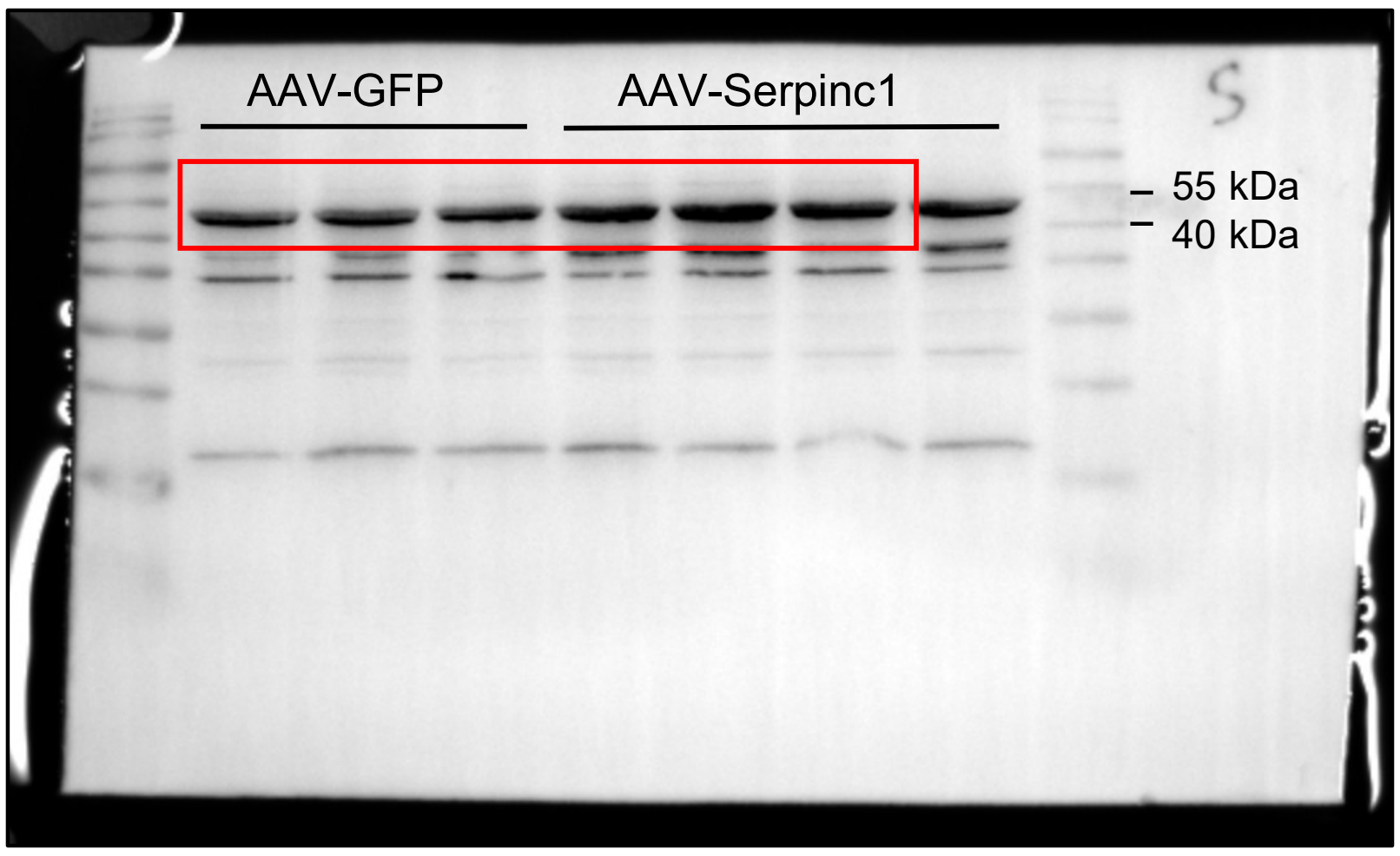

Anti-ATⅢ

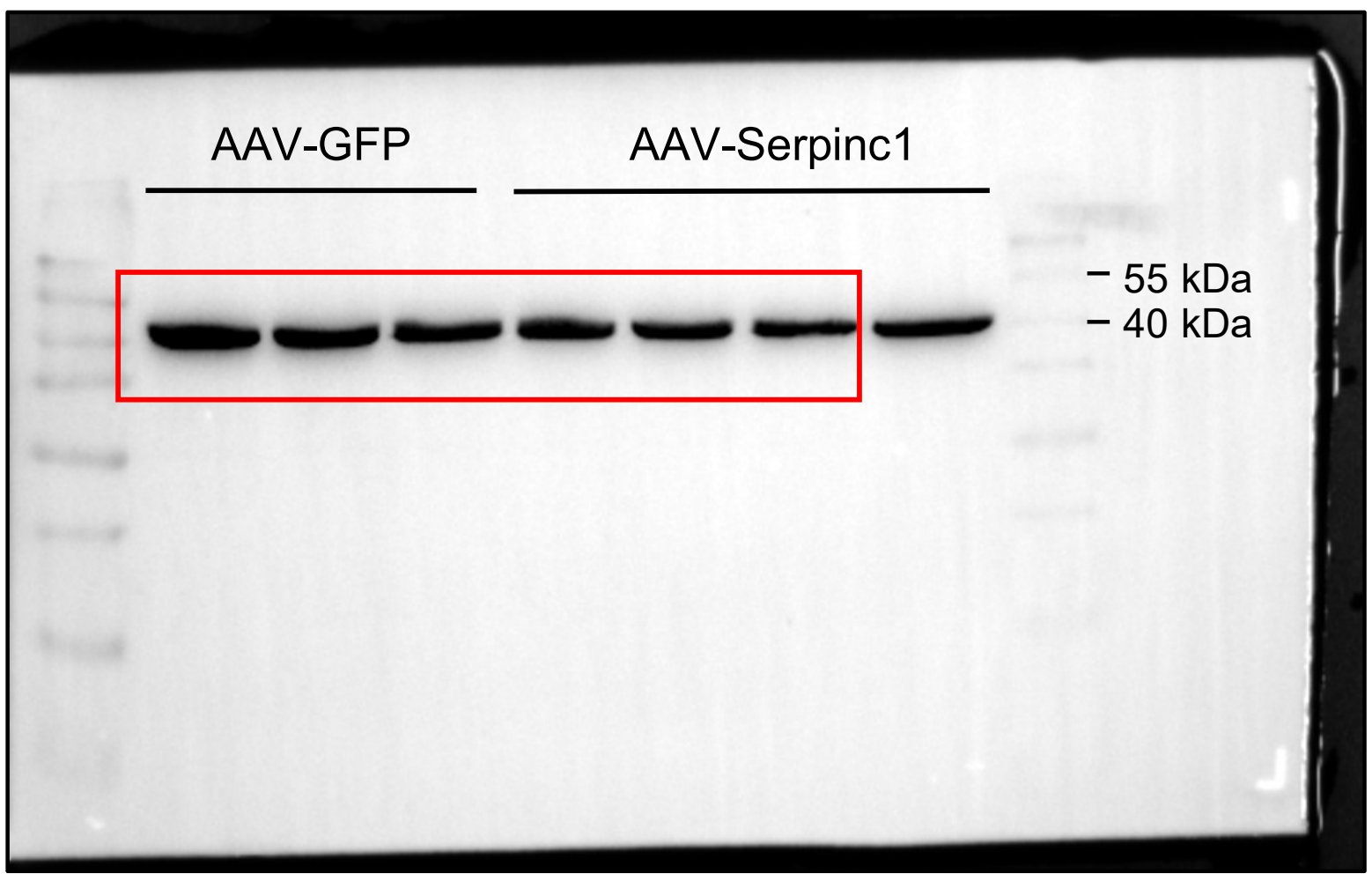

Anti-Actin

Full unedited gel for Supplementary Figure 4

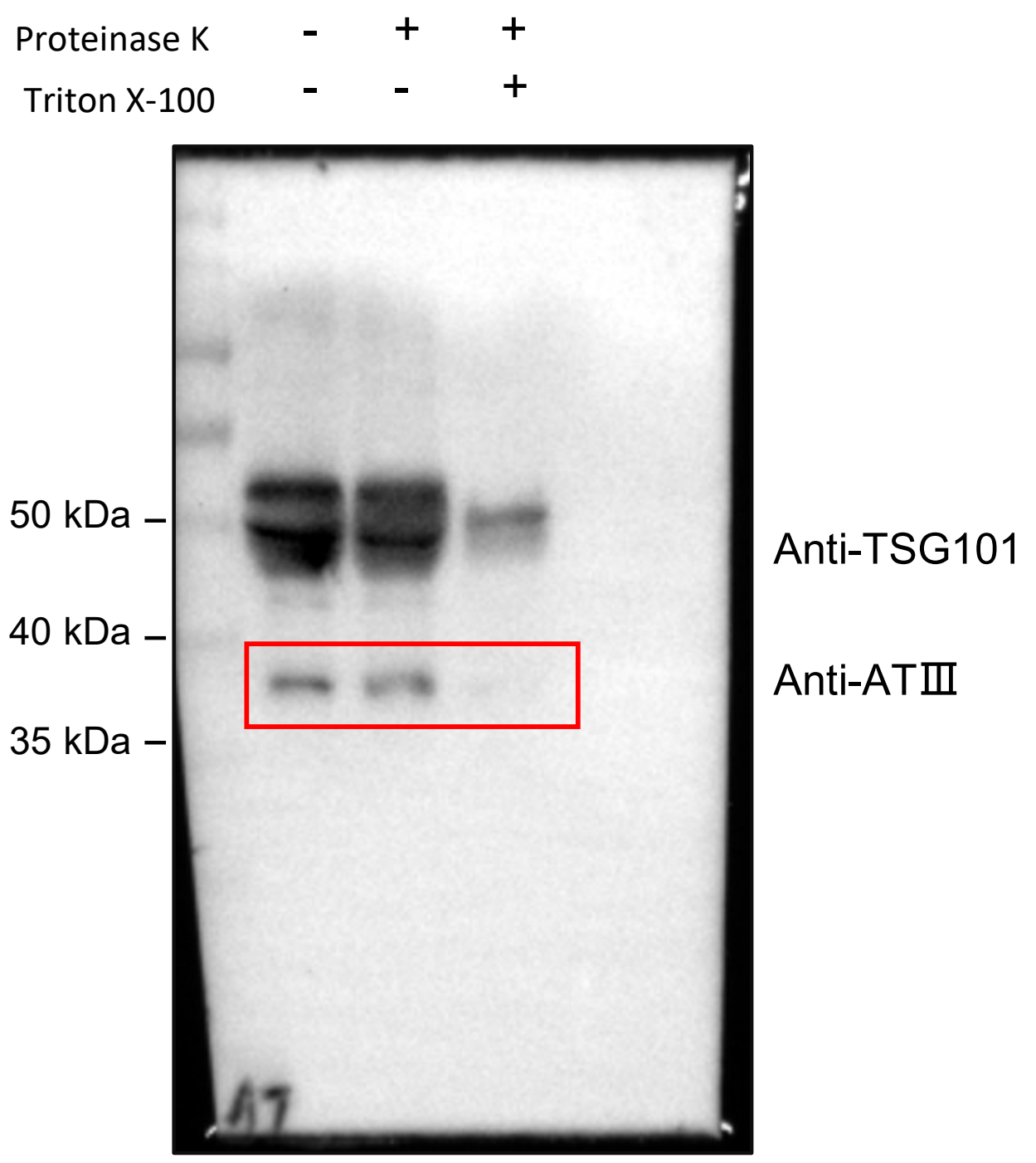

Long exposure

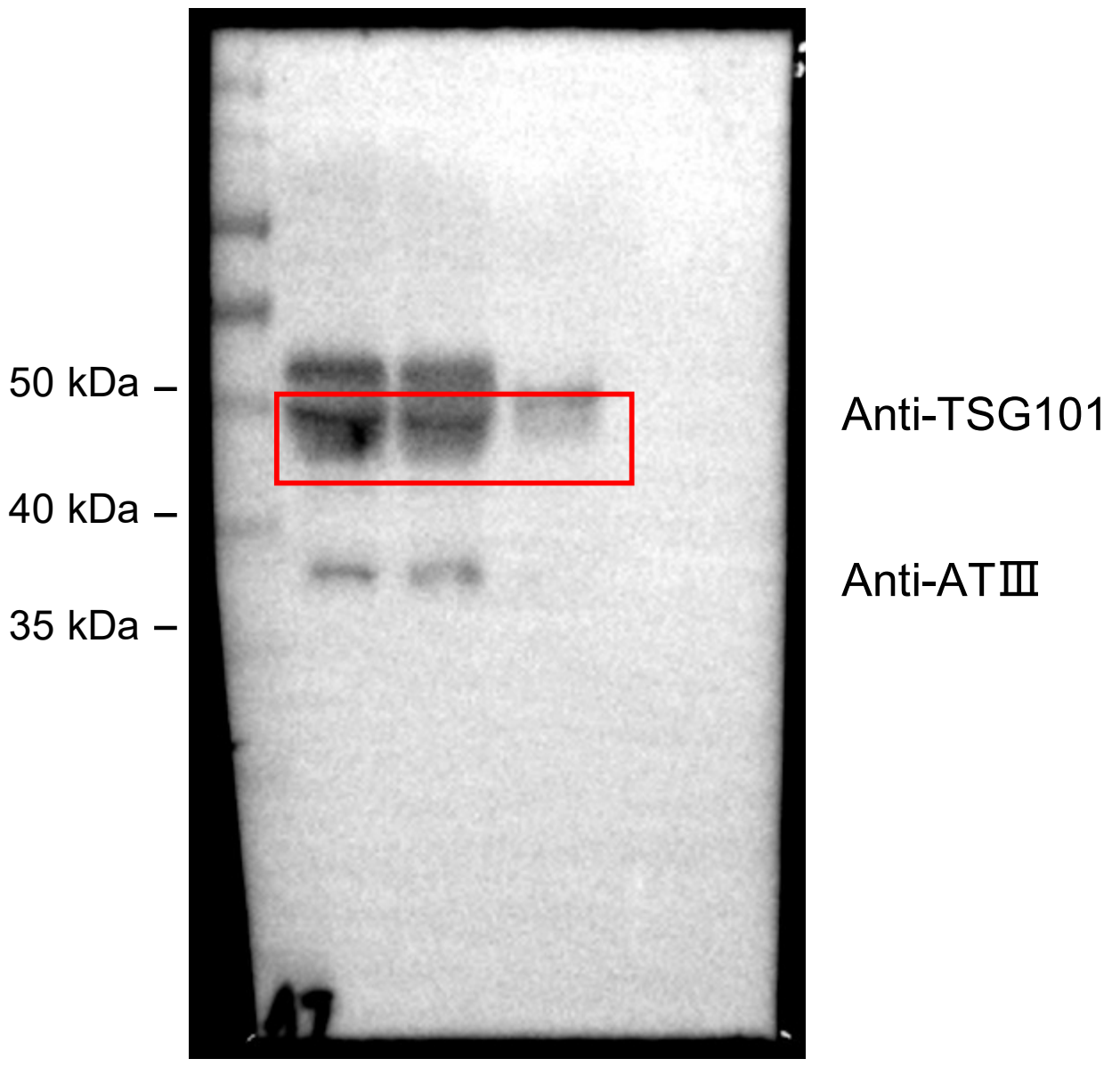

Short exposure

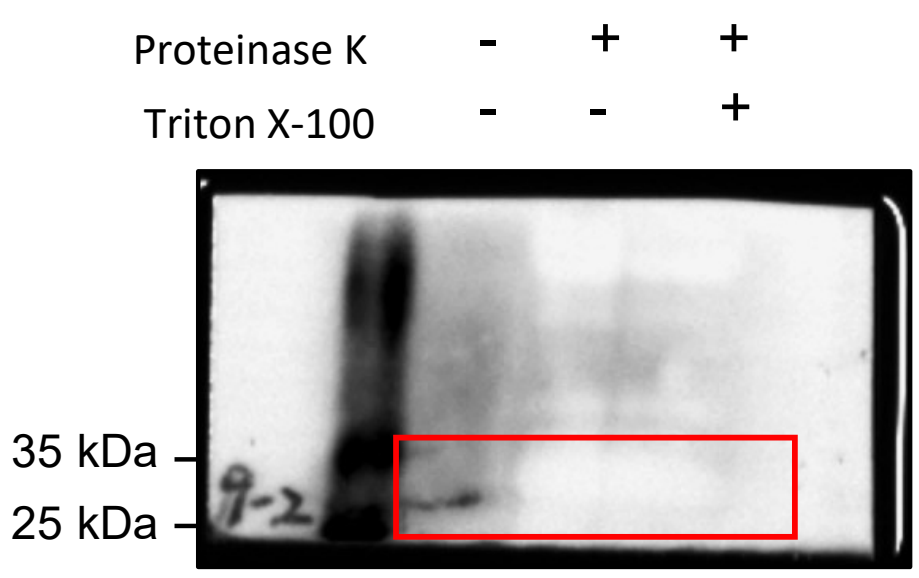

Anti-CD9

Full unedited gel for Supplementary Figure 5

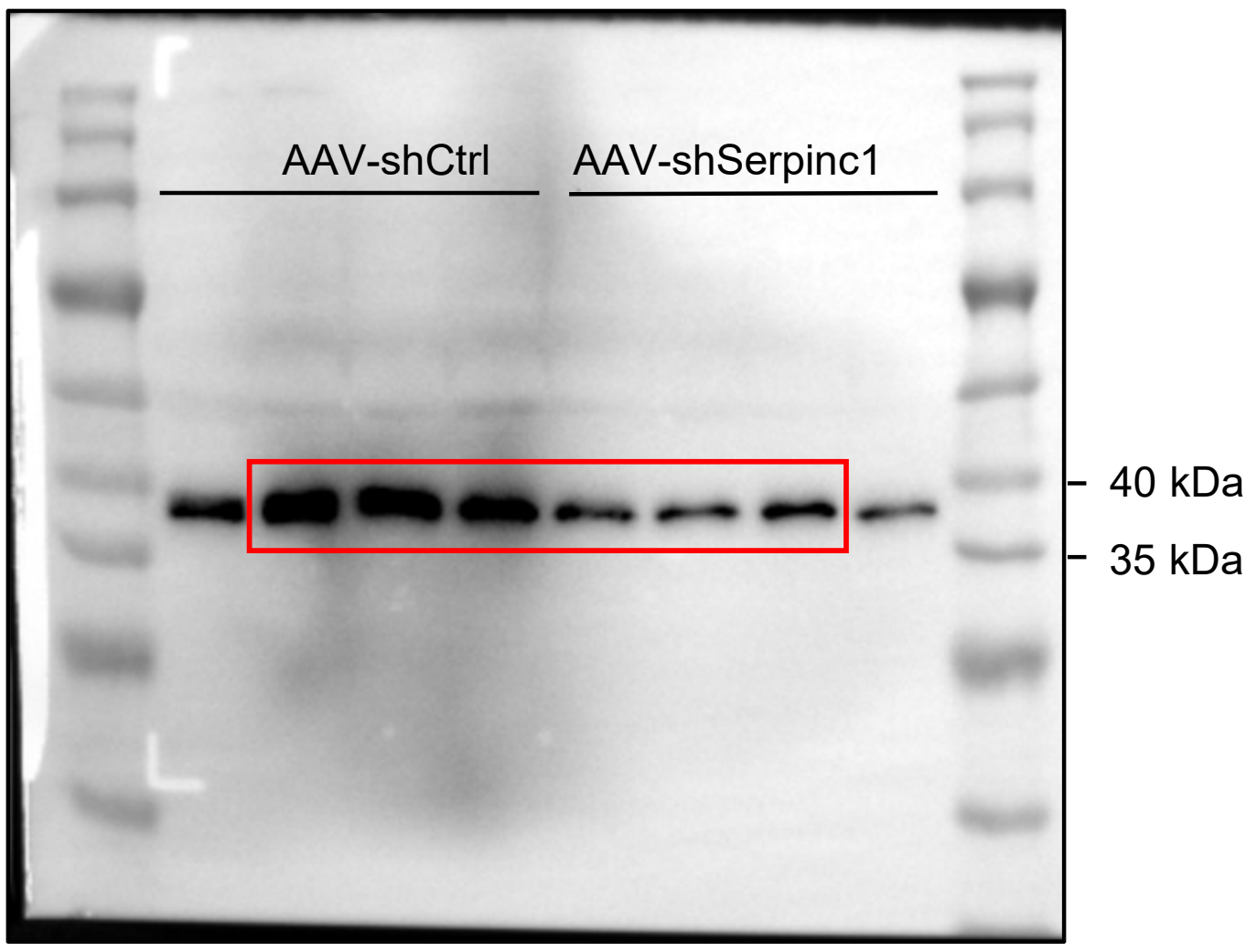

Anti-ATⅢ

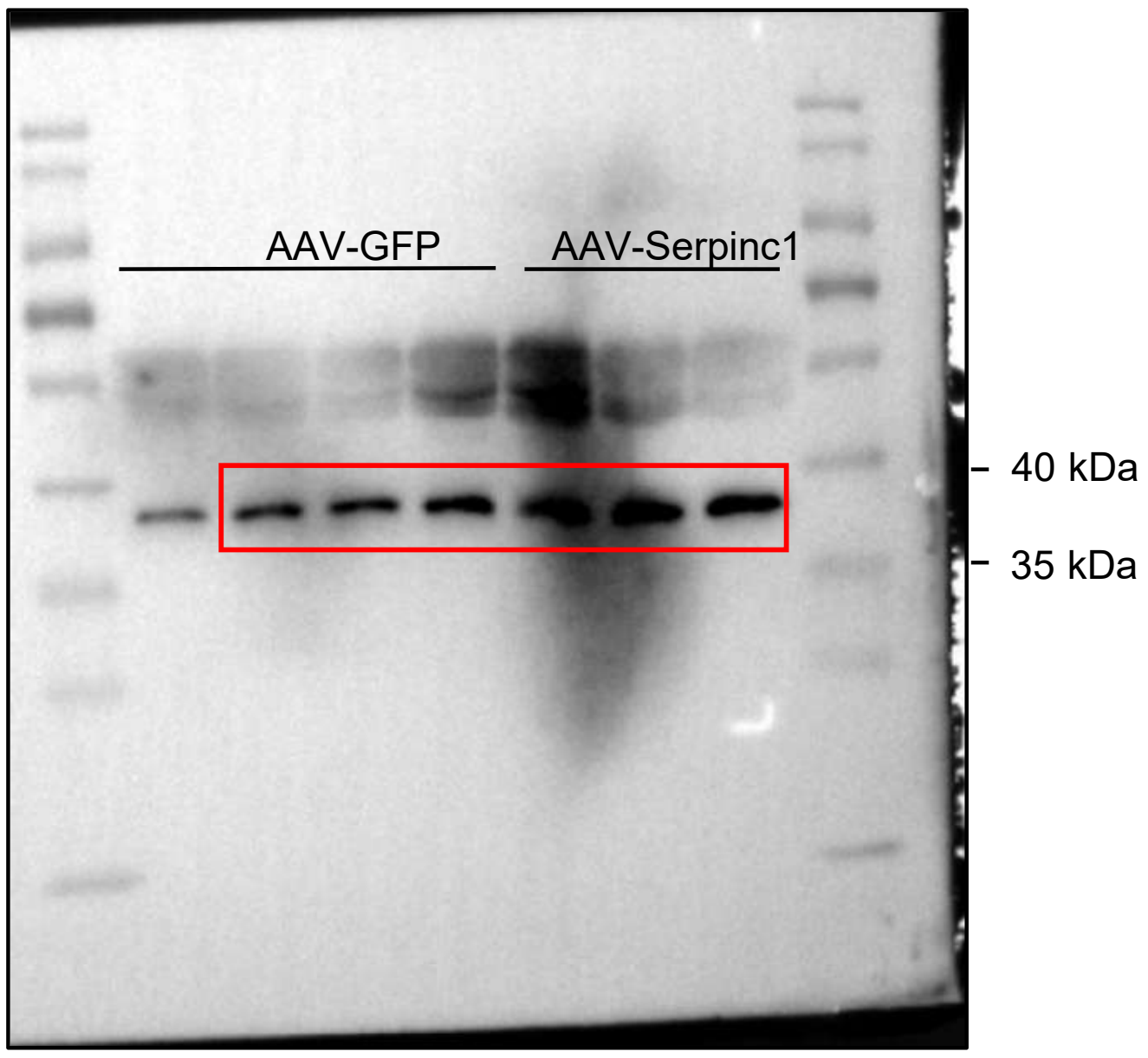

Anti-ATⅢ

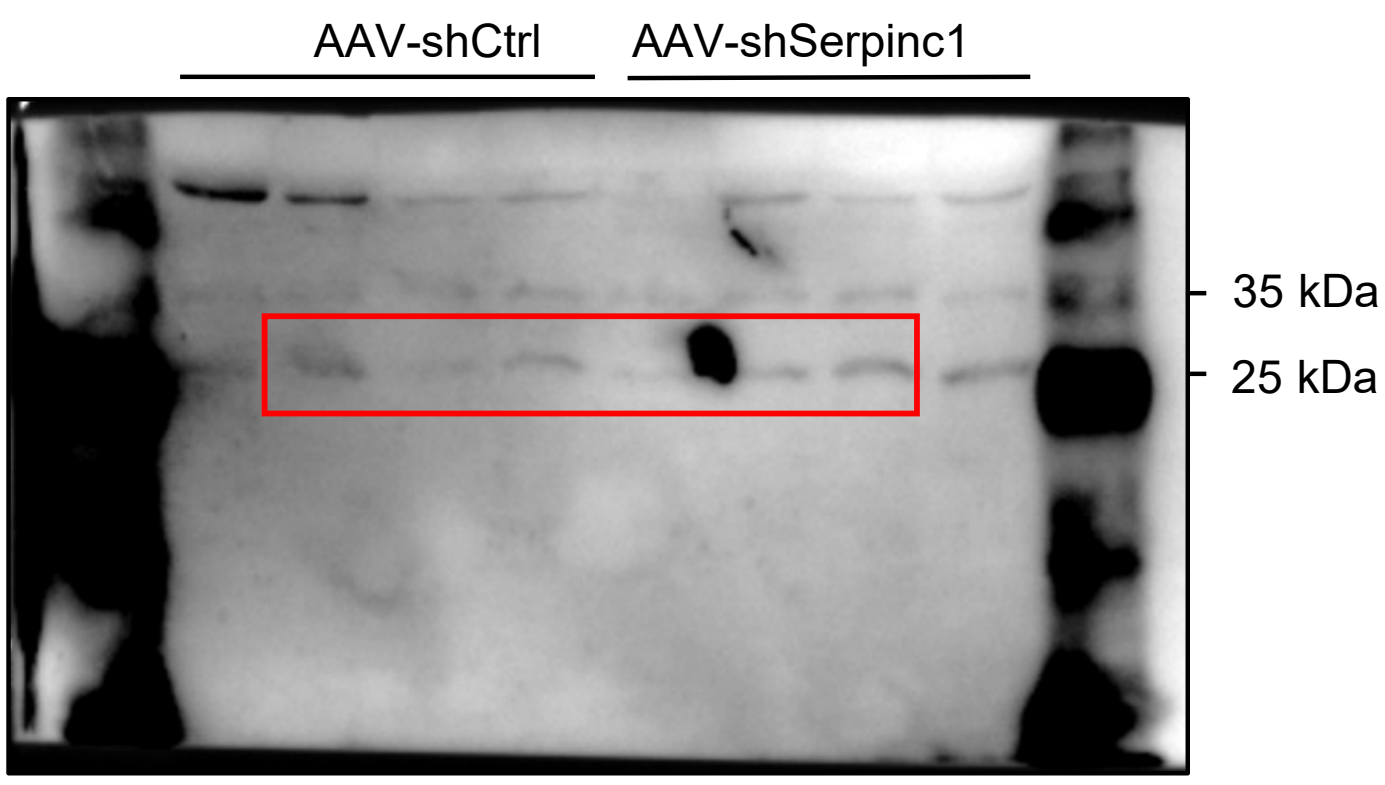

Anti-CD9

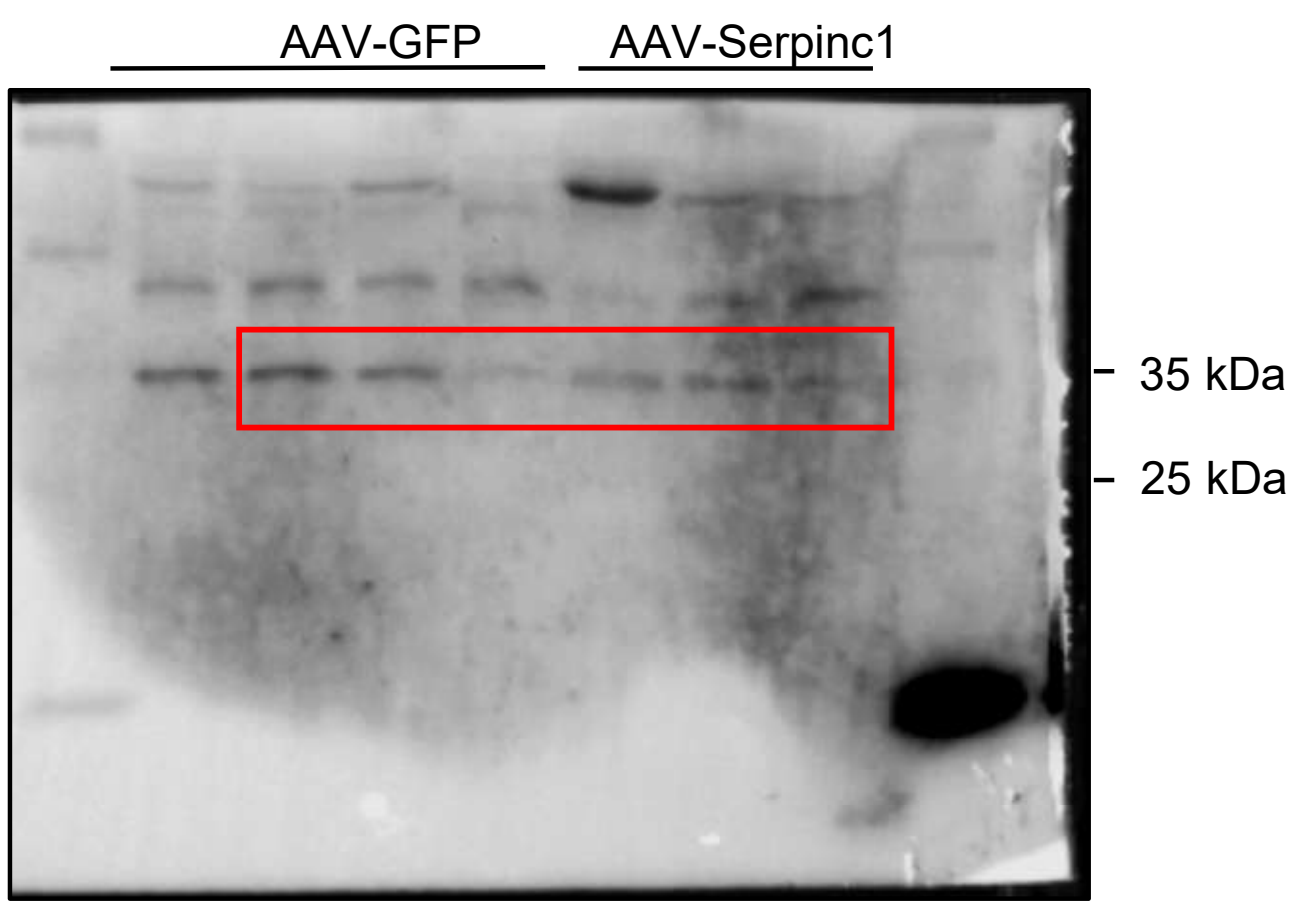

Anti-CD9
